# Supplementary material for: Cell Competition Drives the Growth of Intestinal Adenomas in Drosophila
Source: Curr Biol. 2016 Feb 22;26(4):428–38. doi: 10.1016/j.cub.2015.12.043 (PMC4771920; doi:10.1016/j.cub.2015.12.043)
Supplement: Document S1. Supplemental Experimental Procedures and Figures S1–S6 [file mmc1.pdf]

Current Biology, Volume *26*

Supplemental Information

Cell Competition Drives the Growth of Intestinal Adenomas in *Drosophila*

Saskia J.E. Suijkerbuijk, Golnar Kolahgar, Iwo Kucinski, and Eugenia Piddini

Supplemental Figures and Legends

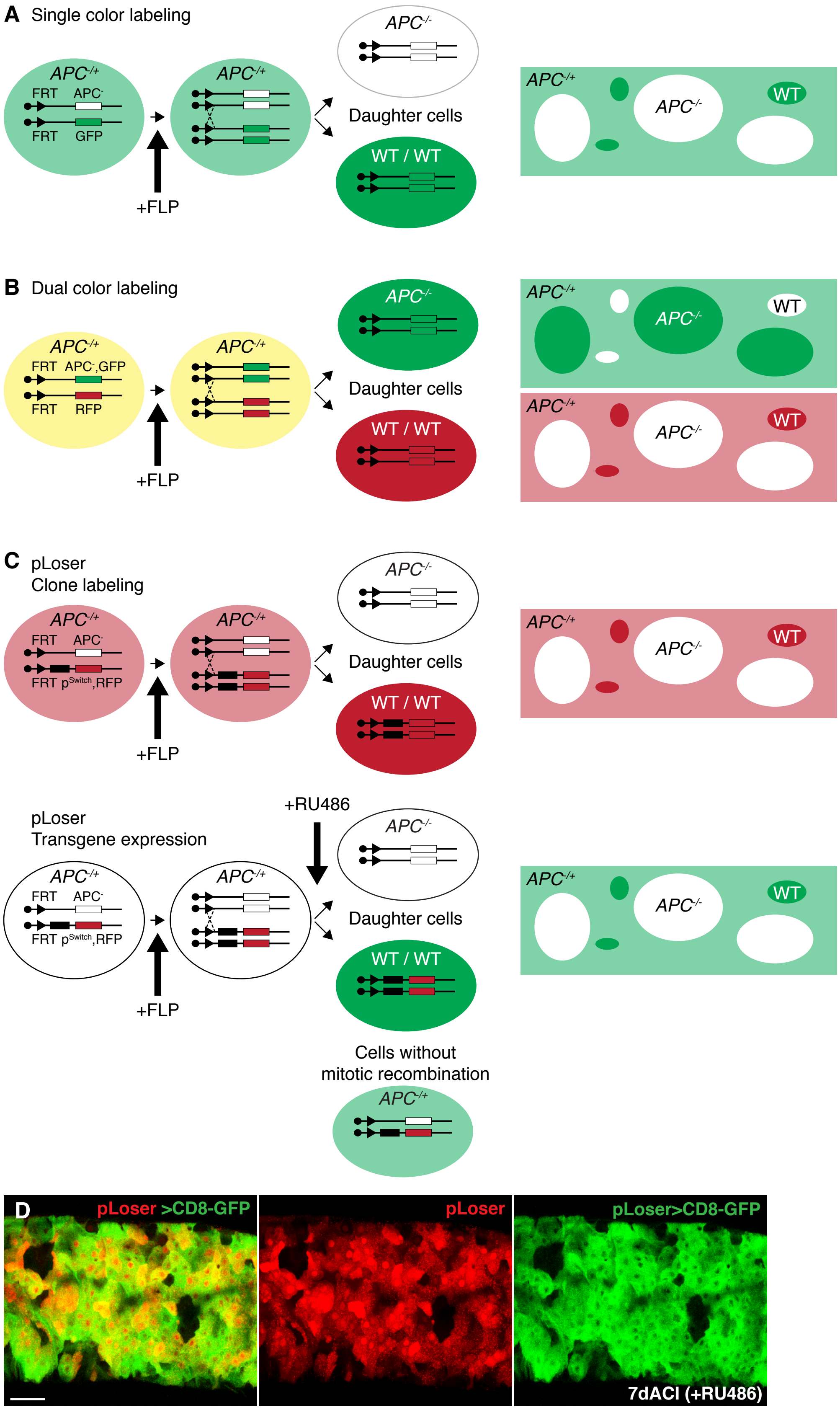

Figure S1, Introduction to single and dual color lineage tracing, Related to Experimental Procedures

A-B) Schematic representation of single (A) and dual (B) color lineage tracing. Heat-shock induced expression of FLP causes mitotic recombination in stem cells that are heterozygous *APC* mutant and express one copy of GFP (A) or one copy of GFP and one copy of RFP (B). This results in the generation of daughter cells that are *APC*$^{-/-}$ (top) or WT/WT (bottom). Clones of cells that are generated by single color labeling (A) are marked by absence of GFP (*APC*$^{-/-}$) or two copies of GFP (WT). Clones of cells that are generated by dual color labeling (B) are marked by absence of RFP and two copies of GFP (*APC*$^{-/-}$) or absence of GFP and two copies of RFP (WT). C) Schematic representation of single color lineage tracing combined with pLoser based transgene expression in host cells. Heat-shock induced expression of FLP causes mitotic recombination in stem cells that are heterozygous *APC* mutant and express one copy of RFP and a RU486 (mifepristone)-inducible GeneSwitch. This results in the generation of daughter cells that are *APC*$^{-/-}$ (top) or WT/WT (bottom). Clones of cells that are generated by pLoser single color labeling are marked by absence of RFP and lack expression of the GeneSwitch. D) Posterior midguts harboring *hs*-FLP induced WT clones marked by absence of RFP. Expression of CD8-GFP was induced by activation of the pLoser GeneSwitch in cells surrounding clones (200μM RU486). Note that absence of RFP directly correlates to absence of GFP induction.

Genotype:

D) *hs-FLP; UAS-CD8-GFP; FRT82B, pLoser, Ubi-RFP-nls / FRT82B*

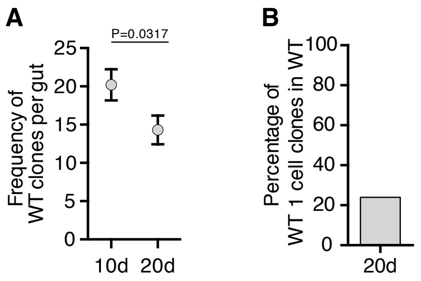

Figure S2, *APC*$^{-/-}$- induced cell competition causes attrition of healthy tissue, Related to Figure 2

A) Frequency of WT clones per midgut from guts containing *APC*$^{-/-}$ clones dissected 10d or 20d ACI (left n=231 and right n=187 clones, ±SEM, P-value is displayed above graph, Mann-Whitney test). B) Percentage of 1-cell clones across the whole population of WT clones in control posterior midguts 20d ACI (n=50 clones).

Genotypes:

A) *hs-FLP; pSwitch*^{all} */+; FRT82B, APC2*^{G10}*, APC1*^{Q8}*, Ubi-GFP/ FRT82B, Ubi-RFP-nls*

B) *hs-FLP;; FRT82B, Ubi-GFP/ FRT82B*

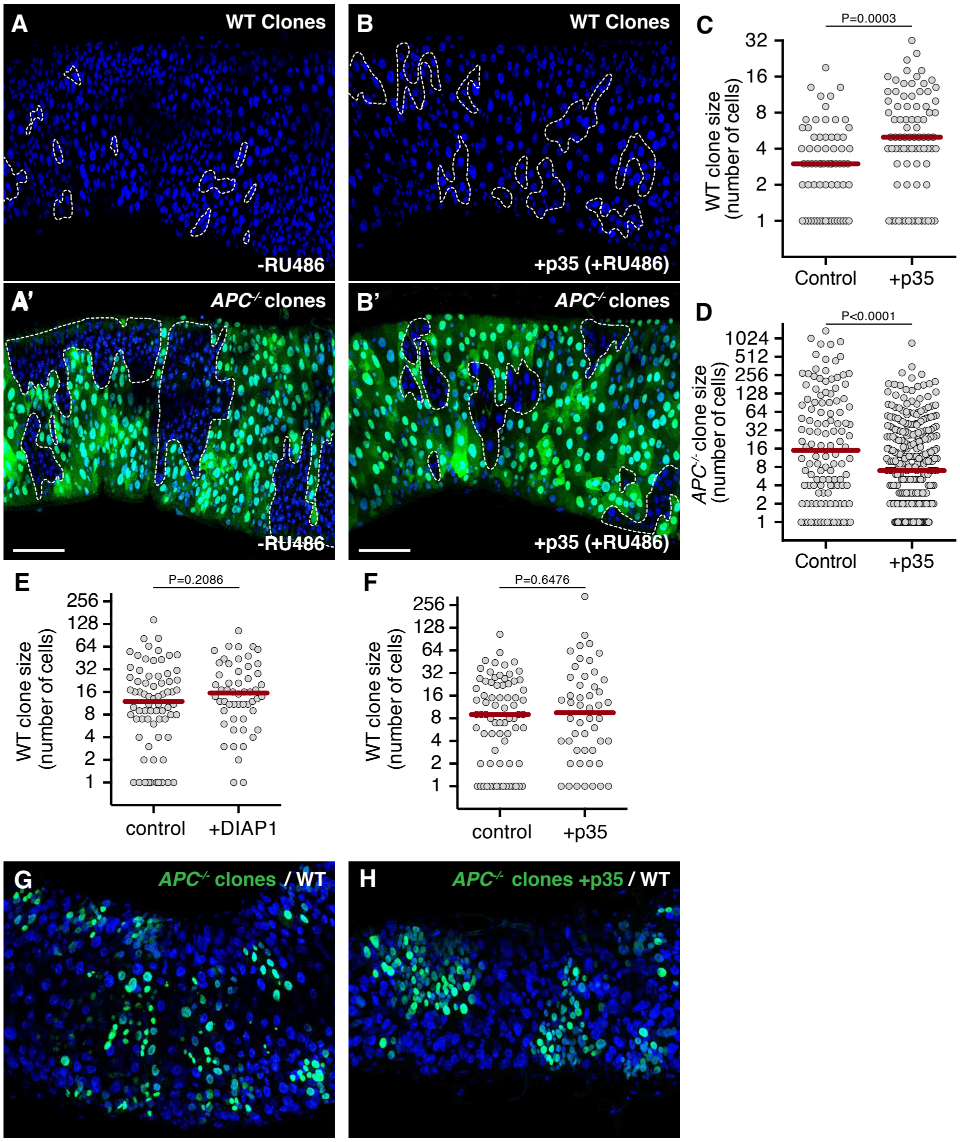

Figure S3, Cell competition fuels tumor growth, Related to Figure 3

A-D) Posterior midguts harboring *hs*-FLP induced WT clones, marked by two copies of GFP (A and B), and *APC*$^{-/-}$ clones, marked by absence of GFP (A' and B') 17dACI. Cell death was blocked by inducible expression of p35 in all ECs and progenitors cells using the GeneSwitch system (+p35, 200μM RU486; B and B'). Control guts (A and A') are of the same genotype as B but were treated with carrier only (-RU486). Graphs in C and D display the distribution of WT (C) or *APC*$^{-/-}$ (D) clone sizes on the y-axis (Log2 scale) from guts with (right) or without (left) p35 expression. Each dot represents one clone and the red bar indicates the median clone size (C left n=67, C right n=82, D left n=122 and D right n=345 clones). E-F) Graphs in E and F display on the y-axis (Log2 scale) the size distribution of *hs*-FLP induced WT clones marked by absence of GFP in WT control posterior midguts (E and F, left graphs) or in WT midguts where cell death was blocked by inducible expression of either DIAP1 (E, right graph) or p35 (F, right graph) in all

ECs and progenitors cells using the GeneSwitch system (40μM RU486). Control guts were treated similarly to experimental guts, but lack the UAS transgene. Each dot represents one clone and the red bar indicates the median clone size (E left n=72, E right n=54, F left n=74 and F right n=50 clones). G-H) Posterior midguts harboring *hs*-FLP induced *APC*$^{-/-}$ clones marked by MARCM-driven expression of GFP, with (H, p35) or without (G, control) additional expression of the cell death inhibitor p35 exclusively in *APC*$^{-/-}$ cells at 17dACI. P-values are displayed above graphs (Mann-Whitney test).

Genotypes:

A-D) *hs-FLP; pSwitch*^{all}*/ UAS-p35; FRT82B, Ubi-GFP/ FRT82B, APC2*^{G10}*, APC1*^{Q8}

E left) *hs-FLP; pSwitch*^{all}*/ GlBc; FRT82B, Ubi-GFP/ FRT82B*

E right) *hs-FLP; pSwitch*^{all}*/ UAS-DIAP1; FRT82B, Ubi-GFP/ FRT82B*

F left) *hs-FLP; pSwitch*^{all}*/ CyO; FRT82B, Ubi-GFP/ FRT82B*

F right) *hs-FLP; pSwitch*^{all}*/ UAS-p35; FRT82B, Ubi-GFP/ FRT82B*

G) *hs-FLP,UAS-GFP-nls,Tub-Gal4;; FRT82B, Tub-Gal80/ FRT82B, APC2*^{G10}*, APC1*^{Q8}

H) *hs-FLP,UAS-GFP-nls,Tub-Gal4; UAS-p35/+; FRT82B,Tub-Gal80/ FRT82B, APC2*^{G10}*, APC1*^{Q8}

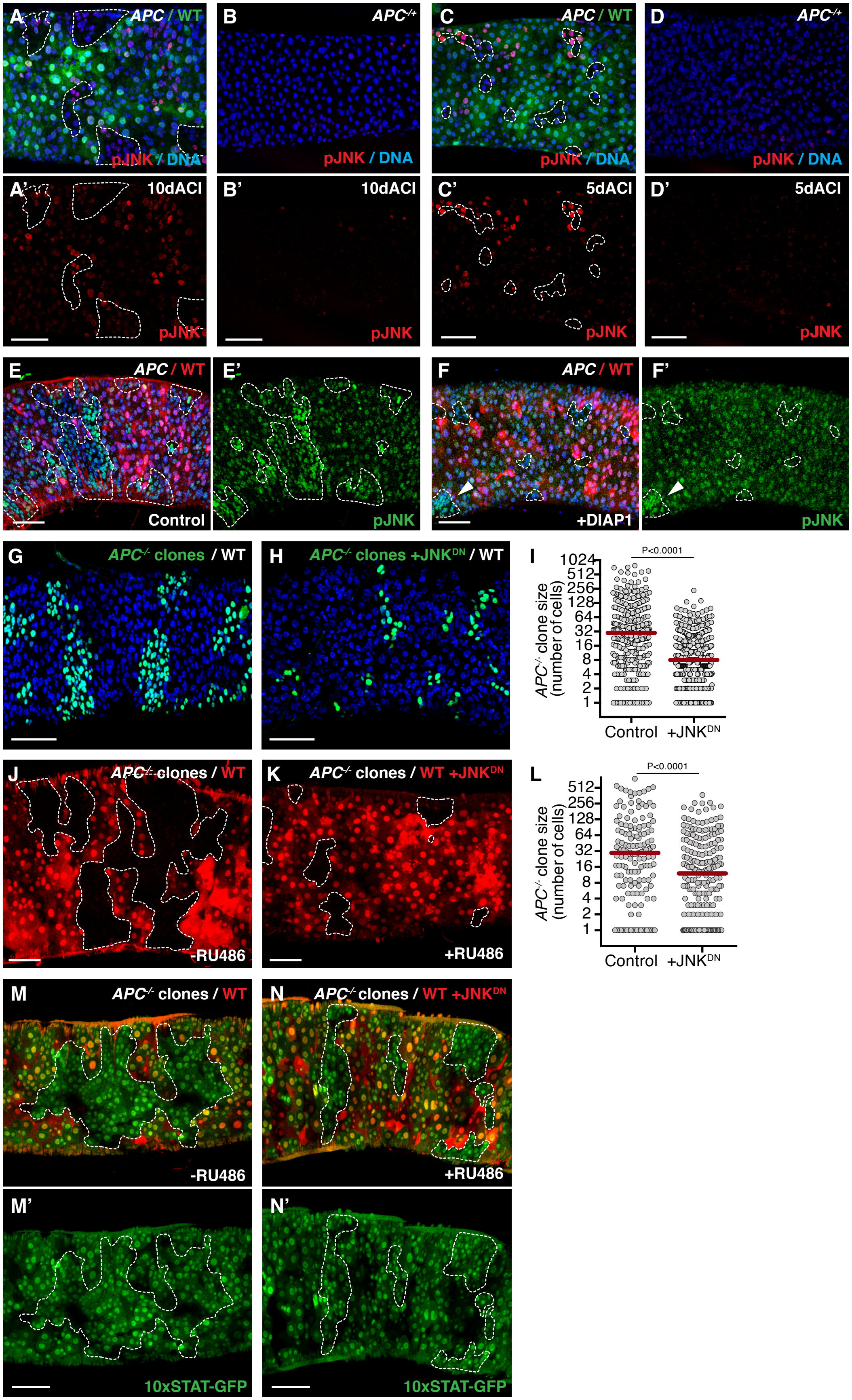
A
APC / WT
pJNK / DNA
B
APC-/+
pJNK / DNA
C
APC / WT
pJNK / DNA
D
APC-/+
pJNK / DNA
A'
10dACI
pJNK
B'
10dACI
pJNK
C'
5dACI
pJNK
D'
5dACI
pJNK
E
APC / WT
Control
E'
pJNK
F
APC / WT
+DIAP1
F'
pJNK
G
APC-/- clones / WT
H
APC-/- clones +JNKDN / WT
I
P<0.0001
APC-/- clone size (number of cells)
1024
512
256
128
64
32
16
8
4
2
1
Control
+JNKDN
J
APC-/- clones / WT
-RU486
K
APC-/- clones / WT +JNKDN
+RU486
L
P<0.0001
APC-/- clone size (number of cells)
512
256
128
64
32
16
8
4
2
1
Control
+JNKDN
M
APC-/- clones / WT
-RU486
N
APC-/- clones / WT +JNKDN
+RU486
M'
10xSTAT-GFP
N'
10xSTAT-GFP

Figure S4, JNK signaling boosts *APC*$^{-/-}$ adenoma growth, Related to Figure 4

A-D) Posterior midguts stained with anti-phospho (active) JNK (pJNK, red). B and D display *APC*$^{-/+}$ heterozygous guts and A and C guts containing *hs*-FLP induced *APC*$^{-/-}$ clones, marked by absence of GFP, dissected 5d (C and D) or 10d (A and B) ACI. E-F) Posterior midguts harboring *hs*-FLP induced *APC*$^{-/-}$ clones marked by absence of RFP dissected 17dACI and stained with anti-phospho (active) JNK (pJNK, green). Cell death was blocked by inducible expression of DIAP1 in all ECs and progenitors cells using the GeneSwitch system (F, +DIAP1, 40μM RU486), control guts were treated similarly to experimental guts, but lack the UAS-DIAP1 transgene (E, control). The arrowhead in F points to an *APC*$^{-/-}$ clone with higher pJNK staining. G-I) Posterior midguts harboring *hs*-FLP induced *APC*$^{-/-}$ clones marked by MARCM-driven expression of GFP, with (H) or without (G) additional expression of JNK^{DN} within the clones at 17dACI. Graph in I displays the distribution of *APC*$^{-/-}$ clone sizes for guts of the same genotypes as in G-H (left n=347 and right n=487 clones). J-L) Posterior midguts harboring *hs*-FLP induced *APC*$^{-/-}$ clones dissected 17dACI. Clones are marked by absence of RFP. JNK signaling was blocked in K by inducible expression of JNK^{DN} in host cells using the GeneSwitch system (+JNK^{DN}, 40μM RU486). Control guts (J) are of the same genotype as K but were treated with carrier only. Graph in L displays the distribution of *APC*$^{-/-}$ clone sizes on the y-axis (Log2 scale) with (right) or without (left) JNK^{DN} expression in host cells. Each dot represents one clone and the red bar indicates the median clone size (left n=148 and right n=214 clones). M-N) Posterior midguts harboring *hs*-FLP induced *APC*$^{-/-}$ clones, marked by absence of RFP, dissected 17dACI. JAK/STAT activity was detected by expression of 10xSTAT-GFP (green). JNK signaling was blocked in N by inducible expression of JNK^{DN} in surrounding cells using the GeneSwitch system starting 7d ACI (+JNK^{DN}, 10days 40μM RU486). Control guts (M) are of the same genotype as N but were treated with carrier only. P-values are displayed above graphs (Mann-Whitney test). Genotypes:

A and C) *hs-FLP;; FRT82B, Ubi-GFP/ FRT82B,* $APC2^{G10}$, $APC1^{Q8}$

B and D) *hs-FLP;; FRT82B,* $APC2^{G10}$, $APC1^{Q8}$*/+*

E) *hs-FLP ; pSwitch*^{all} */ CyO ; diap1-LacZ, FRT82B, Ubi-RFP-nls / FRT82B,* $APC2^{G10}$, $APC1^{Q8}$

F) *hs-FLP ; pSwitch*^{all} */ UAS-DIAP1 ; diap1-LacZ, FRT82B, Ubi-RFP-nls / FRT82B,* $APC2^{G10}$, $APC1^{Q8}$

G, I (left) *hs-FLP, UAS-GFP-nls,Tub-Gal4 ;; FRT82B, Tub-Gal80/ FRT82B,* $APC2^{G10}$,$APC1^{Q8}$

H, I (right) *hs-FLP, UAS-GFP-nls, Tub-Gal4 / UAS-Bsk*^{DN}*;; FRT82B, TubGal80/ FRT82B,* $APC2^{G10}$, $APC1^{Q8}$

J-L) *hs-FLP ; UAS-Puc/+ ; FRT82B, pLoser, Ubi-RFP-nls / FRT82B,*$APC2^{G10}$,$APC1^{Q8}$

M-N) *hs-FLP / UAS-Bsk*^{DN} *; 10xSTAT-GFP/+ ; FRT82B, pLoser, Ubi-RFP-nls / FRT82B,* $APC2^{G10}$, $APC1^{Q8}$

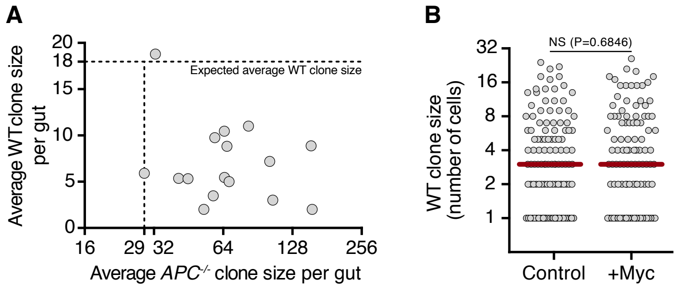

Figure S5, Tumor growth is required for cell competition, Related to Figure 5

A) Graph displaying the average $APC^{-/-}$ clone size per gut (x-axis) plotted against the average WT clone size (y-axis) for clones from guts of the same genotype as in Figure 2B. The dotted horizontal line indicates the expected average WT clone size 17dACI based on the data shown in Figure 2A and 2C, left. The dotted vertical line indicates the extrapolated *$APC^{-/-}$* clone size that is sufficient to induce competition. B) Graph displaying the size distribution on the y-axis (Log2 scale) of WT clones marked by two copies of RFP from guts containing $APC^{-/-}$ clones with (right) or without (left) additional Myc expression throughout host cells (from guts of the same genotype as in Figures 5K (left) L (right)). Each dot represents one clone and the red bar indicates the median clone size (left n=115 and right n=105 clones). The P-value is displayed above graphs (Mann-Whitney test).

Genotypes:

A) *hs-FLP; $pSwitch^{all}$ /+; FRT82B, $APC2^{G10}$, $APC1^{Q8}$, Ubi-GFP/ FRT82B, Ubi-RFP-nls*

B) *hs-FLP ; UAS-Myc/+ ; FRT82B, pLoser, Ubi-RFP-nls / FRT82B, $APC2^{G10}$, $APC1^{Q8}$*

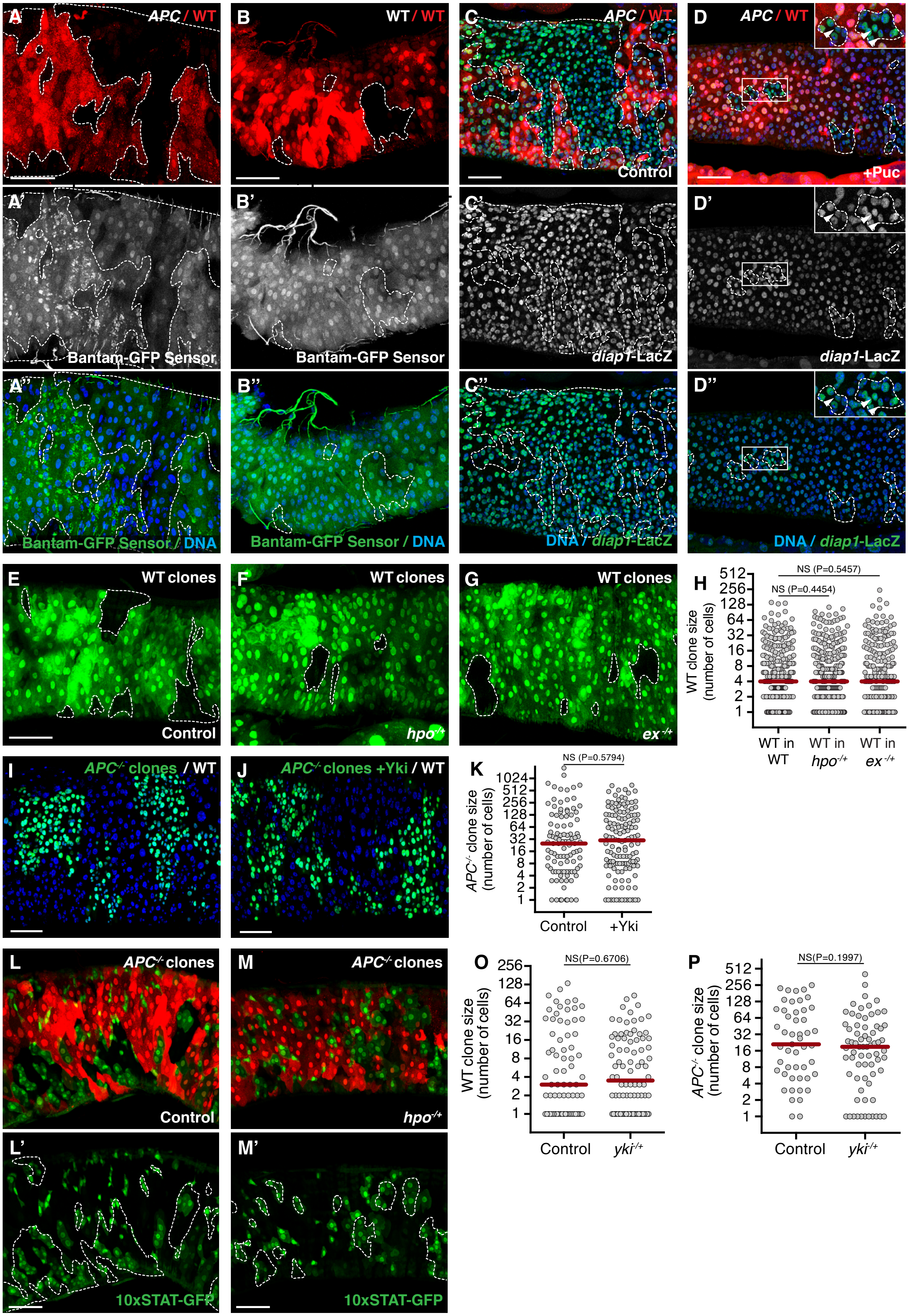
A
APC / WT
B
WT / WT
C
APC / WT
Control
D
APC / WT
+Puc
A'
Bantam-GFP Sensor
B'
Bantam-GFP Sensor
C'
diap1-LacZ
D'
diap1-LacZ
A''
Bantam-GFP Sensor / DNA
B''
Bantam-GFP Sensor / DNA
C''
DNA / diap1-LacZ
D''
DNA / diap1-LacZ
E
WT clones
Control
F
WT clones
hpo-/+
G
WT clones
ex-/+
H
NS (P=0.5457)
NS (P=0.4454)
WT clone size (number of cells)
512
256
128
64
32
16
8
4
2
1
WT in WT
WT in hpo-/+
WT in ex-/+
I
APC-/- clones / WT
J
APC-/- clones +Yki / WT
K
NS (P=0.5794)
APC-/- clone size (number of cells)
1024
512
256
128
64
32
16
8
4
2
1
Control
+Yki
L
APC-/- clones
Control
M
APC-/- clones
hpo-/+
L'
10xSTAT-GFP
M'
10xSTAT-GFP
O
NS(P=0.6706)
WT clone size (number of cells)
256
128
64
32
16
8
4
2
1
Control
yki-/+
P
NS(P=0.1997)
APC-/- clone size (number of cells)
512
256
128
64
32
16
8
4
2
1
Control
yki-/+

Figure S6, Differences in Yki activity determine cell competition potential of *APC*$^{-/-}$ cells, Related to Figure 6

A-B) Posterior midguts with *hs*-FLP induced *APC*$^{-/-}$ (A) or WT (B) clones, marked by absence of RFP, dissected 17d ACI. Yki activity was detected by decreased expression of the Bantam-GFP sensor (A' and B' white and A'' and B'' green). Increased activity was observed in at least one *APC*$^{-/-}$ clone per gut, in about two thirds of *APC*$^{-/-}$ adenoma containing guts (63% n=19). C-D) Posterior midguts harboring *hs*-FLP induced *APC*$^{-/-}$ clones marked by absence of RFP dissected 17dACI. Yki activity was detected by expression of *diap1*-LacZ (C' and D' white and C, D, C'' and D'' green). JNK signaling was blocked by inducible expression of Puckered (D) in all ECs and progenitors cells using the GeneSwitch system (40μM RU486), control guts were treated similarly to experimental guts, but lack UAS transgenes (C, control). Insets in D displays regions containing *APC*$^{-/-}$ clones and arrowheads point at small *APC*$^{-/-}$ mutant cells. E-H) Guts containing WT clones marked by absence of GFP, in control (E), *hpo*$^{-/+}$ (F) or *ex*$^{-/+}$ (G) heterozygous posterior midguts 17dACI. Graph in H displays the distribution of WT clone sizes on the y-axis (Log2 scale) from control (left), *hpo*$^{-/+}$ (middle) or *ex*$^{-/+}$ (right) heterozygous guts (left n=329, middle n=328 and right n=247 clones). I-K) Posterior midguts harboring *hs*-FLP induced *APC*$^{-/-}$ clones marked by MARCM-driven expression of GFP, with (J) or without (I) additional expression of Yki within the clones at 17dACI. Graph in K displays the distribution of *APC*$^{-/-}$ clone sizes for guts of the same genotypes as in I-J (left n=102 and right n=135 clones). L-M) Posterior midguts harboring *hs*-FLP induced *APC*$^{-/-}$ clones, marked by absence of RFP, in a control (L and L') or *hpo*$^{-/+}$ (M and M') heterozygous background, dissected 17dACI. JAK/STAT activity was detected by expression of 10xSTAT-GFP (green). O-P) Graphs display the distribution of simultaneously induced GFP-negative WT (O) or RFP-negative *APC*$^{-/-}$ (P) clone sizes on the y-axis (Log2 scale) from control (left) or *yki*$^{-/+}$ (right) guts. In all graphs each dot represents one clone, the red bars indicate median clone sizes and P-values are displayed above (Mann-Whitney test). Genotypes:

A) *hs-FLP ; Bantam-GFP/ +; FRT82B, Ubi-RFP-nls / FRT82B, APC2*^{G10}*, APC1*^{Q8}

D) *hs-FLP; Bantam-GFP/+; FRT82B, Ubi-RFP-nls/ FRT82B*

C) hs-FLP ; pSwitch^{all} */ CyO ; diap1-LacZ, FRT82B, Ubi-RFP-nls/ FRT82B, APC2*^{G10}*, APC1*^{Q8}

D) hs-FLP; pSwitch^{all}*/ UAS-Puc; diap1-LacZ,FRT82B,Ubi-RFP-nls/ FRT82B,APC2*^{G10}*,APC1*^{Q8}

E&H) *hs-FLP;; FRT82B, Ubi-RFP-nls/ FRT82B, Ubi-GFP*

F&H) *hs-FLP; hpo*$^{42-47}$*/+; FRT82B, Ubi-RFP-nls/ FRT82B, Ubi-GFP*

G&H) *hs-FLP; ex*^{ex1}*/+; FRT82B, Ubi-RFP-nls/ FRT82B, Ubi-GFP*

I& K) *hs-FLP, UAS-GFP-nls,Tub-Gal4; GlBc/+; FRT82B,Tub-Gal80 / FRT82B,APC2*^{G10}*, APC1*^{Q8}

J& K) *hs-FLP,UAS-GFP-nls,Tub-Gal4;UAS-Yki/+;FRT82B,TubGal80/FRT82B,APC2*^{G10}*,APC1*^{Q8}

L) *hs-FLP ; 10xSTAT-GFP/+ ; FRT82B, Ubi-RFP-nls / FRT82B, APC2*^{G10}*, APC1*^{Q8}

M) *hs-FLP ; 10xSTAT-GFP/ hpo*$^{42-47}$ *; FRT82B, Ubi-RFP-nls / FRT82B, APC2*^{G10}*, APC1*^{Q8}

O&P) *hs-FLP; Sp/+; FRT82B, Ubi-RFP-nls / FRT82B, APC2*^{G10}*, APC1*^{Q8}*, Ubi-GFP*

O&P *hs-FLP; Yki*^{B5}*/+; FRT82B, Ubi-RFP-nls / FRT82B, APC2*^{G10}*, APC1*^{Q8}*, Ubi-GFP*

Supplemental Experimental Procedures

Experimental Genotypes

Figure 1:

A:	*hs-FLP ;; FRT82B, Ubi-GFP / FRT82B*
B:	*hs-FLP ;; FRT82B, Ubi-GFP / FRT82B, APC2^{G10}, APC1^{Q8}*
D, C (left):	*hs-FLP ; pSwitch^{all} /+; FRT82B, Ubi-GFP / FRT82B, Ubi-RFP-nls*
E, C (right):	*hs-FLP; pSwitch^{all} /+; FRT82B, APC2^{G10}, APC1^{Q8}, Ubi-GFP / FRT82B, Ubi-RFP-nls*
F, H (left):	*hs-FLP ;; FRT82B, Act-Gal4, UAS-CD8-hPARP-Venus / FRT82B*
G,H (right),I:	*hs-FLP;; FRT82B, Act-Gal4, UAS-CD8-hPARP-Venus / FRT82B, APC2^{G10}, APC1^{Q8}*

Figure 2:

A, C (left):	*hs-FLP ; pSwitch^{all} /+; FRT82B, Ubi-GFP / FRT82B, Ubi-RFP-nls*
B, C(right),D:	*hs-FLP ; pSwitch^{all} /+; FRT82B, APC2^{G10},APC1^{Q8}, Ubi-GFP / FRT82B, Ubi-RFP-nls*

Figure 3:

A-D:	*hs-FLP ; pSwitch^{all} / UAS-DIAP1 ; FRT82B, Ubi-GFP / FRT82B, APC2^{G10}, APC1^{Q8}*
E, G (left):	*hs-FLP, UAS-GFP-nls, Tub-Gal4 ; GlBc / + ; FRT82B, Tub-Gal80 / FRT82B, APC2^{G10}, APC1^{Q8}*
F, G (right):	*hs-FLP,UAS-GFP-nls,Tub-Gal4 ; UAS-DIAP1/+ ; FRT82B,Tub-Gal80 / FRT82B,APC2^{G10},APC1^{Q8}*
H-J:	*hs-FLP ; UAS-DIAP1/+ ; FRT82B, pLoser, Ubi-RFP-nls / FRT82B,APC2^{G10},APC1^{Q8}*

Figure 4:

A:	*hs-FLP ;; FRT82B, Ubi-GFP / FRT82B*
B:	*hs-FLP ;; FRT82B, Ubi-GFP / FRT82B, APC2^{G10}, APC1^{Q8}*
C-F:	*hs-FLP ; pSwitch^{all} / UAS-Puc ; FRT82B, Ubi-GFP / FRT82B, APC2^{G10}, APC1^{Q8}*
G, I& J (left):	*hs-FLP, UAS-GFP-nls,Tub-Gal4;; FRT82B, Tub-Gal80 / FRT82B, APC2^{G10}, APC1^{Q8}, Ubi-GFP*
H, I& J(right):	*hs-FLP, UAS-GFP-nls, Tub-Gal4 ; UAS-Puc/ +; FRT82B, TubGal80 / FRT82B, APC2^{G10}, APC1^{Q8}, Ubi-GFP*
K-M:	*hs-FLP ; UAS-Puc/+ ; FRT82B, pLoser, Ubi-RFP-nls / FRT82B,APC2^{G10},APC1^{Q8}*

Figure 5:

A-D:	*hs-FLP; pSwitch^{all} /+; FRT82B, APC2^{G10}, APC1^{Q8}, Ubi-GFP / FRT82B, Ubi-RFP-nls*
E, G (left):	*hs-FLP, UAS-GFP-nls,Tub-Gal4 ;; FRT82B, Tub-Gal80 / FRT82B, APC2^{G10}, APC1^{Q8}, Ubi-GFP*
F, G (right):	*hs-FLP,UAS-GFP-nls,Tub-Gal4; UAS-Puc/+; FRT82B,TubGal80 / FRT82B, APC2^{G10}, APC1^{Q8},Ubi-GFP*
H, J (left):	*hs-FLP,UAS-GFP-nls,Tub-Gal4 ; GlBc / +; FRT82B,Tub-Gal80 / FRT82B,APC2^{G10},APC1^{Q8},Ubi-GFP*
I, J (right):	*hs-FLP, UAS-GFP-nls, Tub-Gal4; UASMyc^{RNAi} / +; FRT82B,TubGal80 / FRT82B,APC2^{G10},APC1^{Q8},Ubi-GFP*
K-M:	*hs-FLP ; UAS-Myc/+ ; FRT82B, pLoser, Ubi-RFP-nls / FRT82B, APC2^{G10}, APC1^{Q8}*

Figure 6:

A:	*hs-FLP;; diap1-LacZ, FRT82B, Ubi-RFP-nls / FRT82B, APC2^{G10}, APC1^{Q8}, Ubi-GFP*
B:	*hs-FLP ; pSwitch^{all} / UAS-DIAP1 ; diap1-LacZ, FRT82B, Ubi-RFP-nls / FRT82B, APC2^{G10}, APC1^{Q8}*
C, F&G(left):	*hs-FLP ;; FRT82B, Ubi-RFP-nls / FRT82B, APC2^{G10}, APC1^{Q8}, Ubi-GFP*
D, F&G(mid):	*hs-FLP ; hpo$^{42-47}$/ + ; FRT82B, Ubi-RFP-nls / FRT82B, APC2^{G10},APC1^{Q8},Ubi-GFP*
E, F&G(right):	*hs-FLP ; ex^{ex1}/ + ; FRT82B, Ubi-RFP-nls / FRT82B, APC2^{G10}, APC1^{Q8}, Ubi-GFP*

***Drosophila* Stocks**

The following fly stocks were used: *hs-FLP;; FRT82B, Ubi-GFP/TM6B, hs-FLP; Sp/CyO; FRT82B, Ubi-RFP-nls/ TM6B* (Daniel StJohnston), *hs-FLP; Sp/CyO; FRT82B, Ubi-GFP/ TM6B, FRT82B (Bloomington), FRT82B, APC2^{G10}, APC1^{Q8}/TM6B* (M.Peifer), *FRT82B, APC2^{G10}, APC1^{Q8} , Ubi-GFP/ TM6B* (recombinant generated for this study), *FRT82B, Act-Gal4, UAS-CD8-hPARP-Venus* (recombinant generated for this study from *Act-Gal4, UAS-mCD8-hPARP-Venus* [S1, S2] and *FRT82B*), *pSwitch^{all}* (Recominant generated from *pSwitch^{AMP}* and *pSwitch^{PC}* [S3], previously described [S1], *hs-FLP, UAS-GFP-nls, Tub-Gal4;; FRT82B, Tub-Gal80 / TM6B, w; UAS-DIAP1/Cyo KrGal4, UAS-GFP; TM2/ TM6, Df YFP* ([S4] P. Meier), *UAS-Puc^{14C}* (E. Martin-Blanco [S5]), *UAS-Bsk^{DN}* (E. Martin-Blanco [S6]), *Bantam-GFP / CyO* (S. Cohen [S7]), *diap1-LacZ, FRT82B, Ubi-RFP-nls* (Recombinant generated for this study from *LacZ-diap1^{j5C8}* (Bloomington [S8, S9]) and *FRT82B, Ubi-RFP-nls*), *UAS-p35 ; TM2 / TM6B* [S10], *FRT42D, hpo$^{42-47}$/ CyO* (I. Palacios [S9]), *FRT42D, yki^{B5}/ CyO* (I. Palacios [S8]), *w; UAS-yki.GFP^{4-12-1}* (Bloomington), *w; FRT40A, ex^{ex1} / CyO-GFP; hs-FLP, MKRS / TM6B* (J.P. Vincent [S11]), *UAS-myc* (L.A. Baena-Lopez), *UAS-myc^{RNAi}* (VDRC), 10xSTAT-GFP (E. Bach [S12]). The pLoser line (Figure S1C) was generated by recombination of P{Switch2}GSG2326 (Bloomington) and *FRT82B, Ubi-RFP-nls.* The integration site of P{Switch2}GSG2326 was mapped to chromosome 3R by inverse PCR (www.fruitfly.org/about/methods/inverse.pcr) and induction of expression in the midgut epithelium upon RU486 (mifepristone) feeding was validated with UAS-CD8-mGFP (Figure S1D).

Antibodies:

Mouse anti-Delta (DSHB, C594.9B) 1/1000, mouse anti-Prospero (DSHB, MR1A) 1/50, chicken anti-GFP (Abcam, ab13970) 1/500, rabbit or mouse anti-Cleaved human PARP (Abcam ab2317, 1/100 and Abcam ab110315, 1/500 respectively), rabbit anti-pJNK pTPpY (Promega V793B) 1/500, chicken anti-β-galactosidase (Abcam ab9361) 1/500 and rabbit anti-phospho Histone H3 Ser10 (Cell Signaling 9701) 1/1000. Secondary antibodies used were coupled to Alexa488, Alexa555 or Alexa633 or Cy5 (Molecular Probes). Nuclei were counterstained with DAPI or Hoechst 33342.

Generation of mitotic clones

For clone generation, single stem cell-derived clones were generated by mitotic recombination, using the FLP/FRT system [S13]. One to two days after eclosion, fertilized female flies were heat-shocked in a water bath at 37°C for 10 minutes and then reared at 25°C. Clones were induced

sparsely to minimize clone fusion, except for Figure 6A, where flies were heat-shocked in a water bath at 37°C for 30 minutes, with the intention of generating large clones. Flies were aged up to a maximum of 20 days ACI, to avoid ageing effects, which disrupt tissue homeostasis.

Confocal Acquisition and image analysis

Samples were imaged with Leica SP5 inverted or Leica SP8 upright confocal microscopes, using a 40x 1.3 NA PL Apo or 40x/1.3 HC PL Apo CS2 Oil objective respectively. All images were taken as z-stacks of 1µm sections in the posterior midgut region immediately anterior to the hindgut (these corresponds to the regions P4 in [S14] or region R5 in [S15]. Image processing, analysis and 3D reconstruction were done with Volocity (Perkin Elmer, version 6.3) and Photoshop (Adobe version CS6).

Cell counting

All quantifications were done throughout the volume of 3D reconstructions of z-stacks using Volocity (Perkin Elmer, version 6.3). Quantifications of cell numbers were done manually. Clone sizes were calculated as the number of DAPI positive cells per clone in the 3D volume. To count cells around clones ("near" in Figure 1H) we counted all cells surrounding a clone within two cell diameters in the 3D volume. To characterize cells not adjacent to clones ("far" in Figure 1FH, we counted cells that were not in within two cell diameters of a clone or at the edge of the image. For quantification of WT clones in the absence of tumors (Figure 5F and 5G, right graph) we only included samples in which inhibition of JNK signaling had efficiently reduced tumor growth.

Statistical tests

Statistical analyses were done using Prism (GraphPad, version 6.0 for Mac OS X). P-values were determined using the non-parametric Mann-Whitney test throughout, except for Figures 2D, 4I-J where a Fisher's exact contingency test or t test was used respectively.

Supplemental References

S1. Kolahgar, G., Suijkerbuijk, S. J. E., Kucinski, I., Poirier, E. Z., Mansour, S., Simons, B. D., and Piddini, E. (2015). Cell competition modifies adult stem cell and tissue population dynamics in a JAK- STAT dependent manner. Developmental Cell, 1–14.

S2. Williams, D. W., Kondo, S., Krzyzanowska, A., Hiromi, Y., and Truman, J. W. (2006). Local caspase activity directs engulfment of dendrites during pruning. Nat Neurosci *9*, 1234–1236.

S3. Mathur, D., Bost, A., Driver, I., and Ohlstein, B. (2010). A transient niche regulates the specification of Drosophila intestinal stem cells. Science *327*, 210–213.

S4. Hay, B. A., Wassarman, D. A., and Rubin, G. M. (1995). Drosophila homologs of baculovirus inhibitor of apoptosis proteins function to block cell death. Cell *83*, 1253–1262.

S5. Martín-Blanco, E., Gampel, A., Ring, J., Virdee, K., Kirov, N., Tolkovsky, A. M., and Martinez-Arias, A. (1998). puckered encodes a phosphatase that mediates a feedback loop regulating JNK activity during dorsal closure in Drosophila. Genes Dev. *12*, 557–570.

S6. Adachi-Yamada, T., Nakamura, M., Irie, K., Tomoyasu, Y., Sano, Y., Mori, E., Goto, S., Ueno, N., Nishida, Y., and Matsumoto, K. (1999). p38 mitogen-activated protein kinase can be involved in transforming growth factor beta superfamily signal transduction in Drosophila wing morphogenesis. Molecular and Cellular Biology *19*, 2322–2329.

S7. Brennecke, J., Hipfner, D. R., Stark, A., Russell, R. B., and Cohen, S. M. (2003). bantam encodes a developmentally regulated microRNA that controls cell proliferation and regulates the proapoptotic gene hid in Drosophila. Cell *113*, 25–36.

S8. Huang, J., Wu, S., Barrera, J., Matthews, K., and Pan, D. (2005). The Hippo signaling pathway coordinately regulates cell proliferation and apoptosis by inactivating Yorkie, the Drosophila Homolog of YAP. Cell *122*, 421–434.

S9. Wu, S., Huang, J., Dong, J., and Pan, D. (2003). hippo encodes a Ste-20 family protein kinase that restricts cell proliferation and promotes apoptosis in conjunction with salvador and warts. Cell *114*, 445–456.

S10. Hay, B. A., Wolff, T., and Rubin, G. M. (1994). Expression of baculovirus P35 prevents cell death in Drosophila. Development *120*, 2121–2129.

S11. Hamaratoglu, F., Willecke, M., Kango-Singh, M., Nolo, R., Hyun, E., Tao, C., Jafar-Nejad, H., and Halder, G. (2006). The tumour-suppressor genes NF2/Merlin and Expanded act through Hippo signalling to regulate cell proliferation and apoptosis. Nature Cell Biology *8*, 27–36.

S12. Bach, E. A., Ekas, L. A., Ayala-Camargo, A., Flaherty, M. S., Lee, H., Perrimon, N., and Baeg, G.-H. (2007). GFP reporters detect the activation of the Drosophila JAK/STAT pathway in vivo. Gene Expr. Patterns *7*, 323–331.

S13. Xu, T., and Rubin, G. M. (1993). Analysis of genetic mosaics in developing and adult Drosophila tissues. Development *117*, 1223–1237.

S14. Marianes, A., and Spradling, A. C. (2013). Physiological and stem cell compartmentalization within the Drosophila midgut. Elife *2*, e00886.

S15. Buchon, N., Osman, D., David, F. P. A., Fang, H. Y., Boquete, J.-P., Deplancke, B., and Lemaitre, B. (2013). Morphological and Molecular Characterizationof Adult Midgut Compartmentalization in Drosophila. Cell Rep *3*, 1725–1738.
