# Supplementary material for: Cell Competition Drives the Growth of Intestinal Adenomas in Drosophila
Source: Curr Biol. 2016 Feb 22;26(4):428–38. doi: 10.1016/j.cub.2015.12.043 (PMC4771920; doi:10.1016/j.cub.2015.12.043)
Supplement: Document S2. Article plus Supplemental Information [file mmc2.pdf]

Article

Current Biology

Cell Competition Drives the Growth of Intestinal Adenomas in *Drosophila*

Graphical Abstract

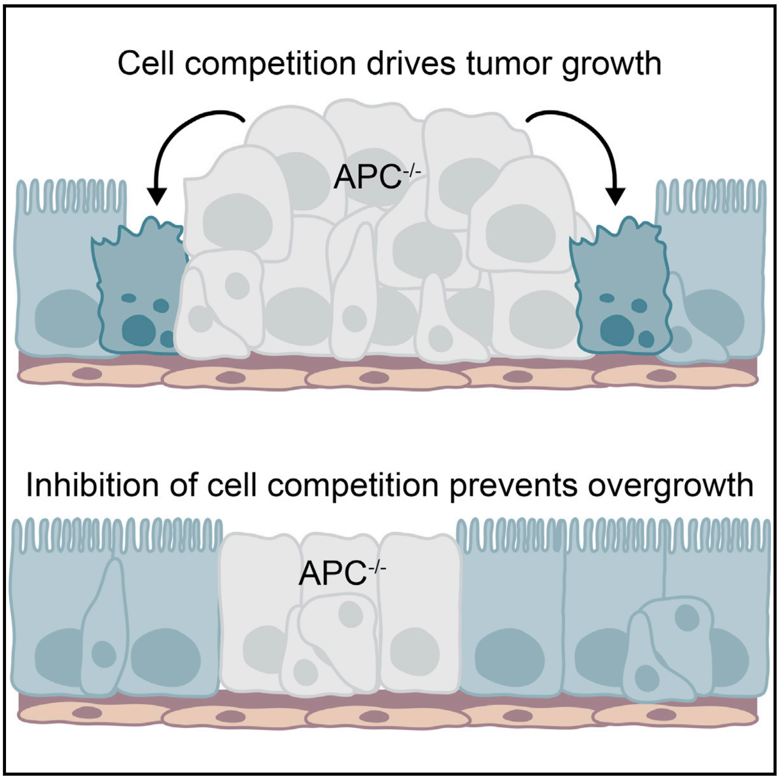

Highlights

- In the fly gut *APC*$^{-/-}$ tumors kill surrounding cells and cause loss of healthy tissue
- Tumor-host cell competition fuels tumor growth
- JNK signaling boosts *APC*$^{-/-}$ adenoma growth and drives cell competition
- Differences in Yki activity determine the cell competition potential of *APC*$^{-/-}$ cells

Authors

Saskia J.E. Suijkerbuijk, Golnar Kolahgar, Iwo Kucinski, Eugenia Piddini

Correspondence

e.piddini@gurdon.cam.ac.uk

In Brief

Suijkerbuijk et al. show that growing tumors in the adult *Drosophila* posterior midgut kill surrounding cells and cause host tissue attrition through a process called cell competition. Inhibition of cell competition prevents adenoma growth, indicating that it is an essential driver of tumor growth in this tissue.

Suijkerbuijk et al., 2016, Current Biology *26*, 428–438
February 22, 2016 ©2016 The Authors
http://dx.doi.org/10.1016/j.cub.2015.12.043

CellPress

Current Biology
Article

Cell Competition Drives the Growth of Intestinal Adenomas in *Drosophila*

Saskia J.E. Suijkerbuijk,[1,2] Golnar Kolahgar,[1] Iwo Kucinski,[1] and Eugenia Piddini[1,*]
[1]The Wellcome Trust/Cancer Research UK Gurdon Institute, University of Cambridge, Tennis Court Road, Cambridge CB2 1QN, UK
[2]Present address: Hubrecht Institute (KNAW), Uppsalalaan 8, 3584 CT Utrecht, the Netherlands
*Correspondence: e.piddini@gurdon.cam.ac.uk
http://dx.doi.org/10.1016/j.cub.2015.12.043
This is an open access article under the CC BY license (http://creativecommons.org/licenses/by/4.0/).

SUMMARY

Tumor-host interactions play an increasingly recognized role in modulating tumor growth. Thus, understanding the nature and impact of this complex bidirectional communication is key to identifying successful anti-cancer strategies. It has been proposed that tumor cells compete with and kill neighboring host tissue to clear space that they can expand into; however, this has not been demonstrated experimentally. Here we use the adult fly intestine to investigate the existence and characterize the role of competitive tumor-host interactions. We show that *APC*$^{-/-}$-driven intestinal adenomas compete with and kill surrounding cells, causing host tissue attrition. Importantly, we demonstrate that preventing cell competition, by expressing apoptosis inhibitors, restores host tissue growth and contains adenoma expansion, indicating that cell competition is essential for tumor growth. We further show that JNK signaling is activated inside the tumor and in nearby tissue and is required for both tumor growth and cell competition. Lastly, we find that *APC*$^{-/-}$ cells display higher Yorkie (YAP) activity than host cells and that this promotes tumor growth, in part via cell competition. Crucially, we find that relative, rather than absolute, Hippo activity determines adenoma growth. Overall, our data indicate that the intrinsic over-proliferative capacity of *APC*$^{-/-}$ cells is not uncontrolled and can be constrained by host tissues if cell competition is inhibited, suggesting novel possible therapeutic approaches.

INTRODUCTION

It is increasingly recognized that tumors do not simply depend on their own proliferative capacity for growth, but instead interact with their environment on multiple levels. For example, the tumor microenvironment can have a growth-enhancing role by inducing a wound healing like pro-proliferative milieu [1] or by recruiting tumor enhancing cancer-associated fibroblasts [2, 3]. However, in certain instances, tumor-host interactions have also been reported to inhibit tumor growth [4]. For example, embryonic environments have been shown to suppress the aggressiveness of multiple cancer cells [5, 6]. In addition, in some contexts, fibroblasts have been shown to limit the growth and malignancy of neoplastic cells [7]. This suggests that understanding how to enhance the tumor suppressive properties of host tissues may help in the fight against cancer.

Reciprocally, it has also been suggested that precancerous lesions and growing tumors could adversely affect the host tissue. Specifically, it has been proposed that tumor cells could kill surrounding normal cells and use this strategy to clear space in which they can expand. This suggestion stems from the observation that in developing tissues, cells with tumor promoting mutations can induce cell death in nearby wild-type cells [8, 9]. In particular, it has been suggested that cancer cells co-opt a form of cell interaction normally present in tissues, known as cell competition [10, 11]. Cell competition was originally discovered in *Drosophila* when it was found that wild-type cells can kill cells with mutations that reduce their fitness and growth potential [12] and has been suggested to act as a quality-control mechanism to preserve tissue function [13, 14]. It was later found that in developing tissues, wild-type cells themselves could be killed via cell competition by mutant cells harboring oncogenic mutations, so called supercompetitor cells [8, 9]. This led to the long-standing hypothesis that tumor-host cell competition might take place and promote tumor formation; however, this has never been tested directly in adult tissues.

The adult *Drosophila* midgut has recently been established as a model system to study adult stem cell behavior, tissue homeostasis, aging, and regeneration [15–17]. This tissue has a high cellular turnover and is maintained by newly differentiated cells produced from intestinal stem cells (ISCs), in a way that is remarkably similar to the mammalian intestine [17]. Importantly, mutations that are involved in cancer have also been found to lead to overgrowth and tumor formation in the fly intestine [18–20], in some cases by niche appropriation [21]. Furthermore, we have recently shown that cell competition is active and plays a role in shaping tissue colonization in this tissue [22]. Overall, these features provide a unique opportunity to combine the power of *Drosophila* genetics and the simplicity of this adult homeostatic tissue to study the role of cell competition in tumor formation.

Here we show that *Drosophila* intestinal tumors compete with and induce elimination of surrounding cells, causing host tissue attrition. Importantly, we demonstrate that preventing cell competition, e.g., by inhibiting cell death, dramatically reduces tumor growth. Thus, by generating an environment permissive

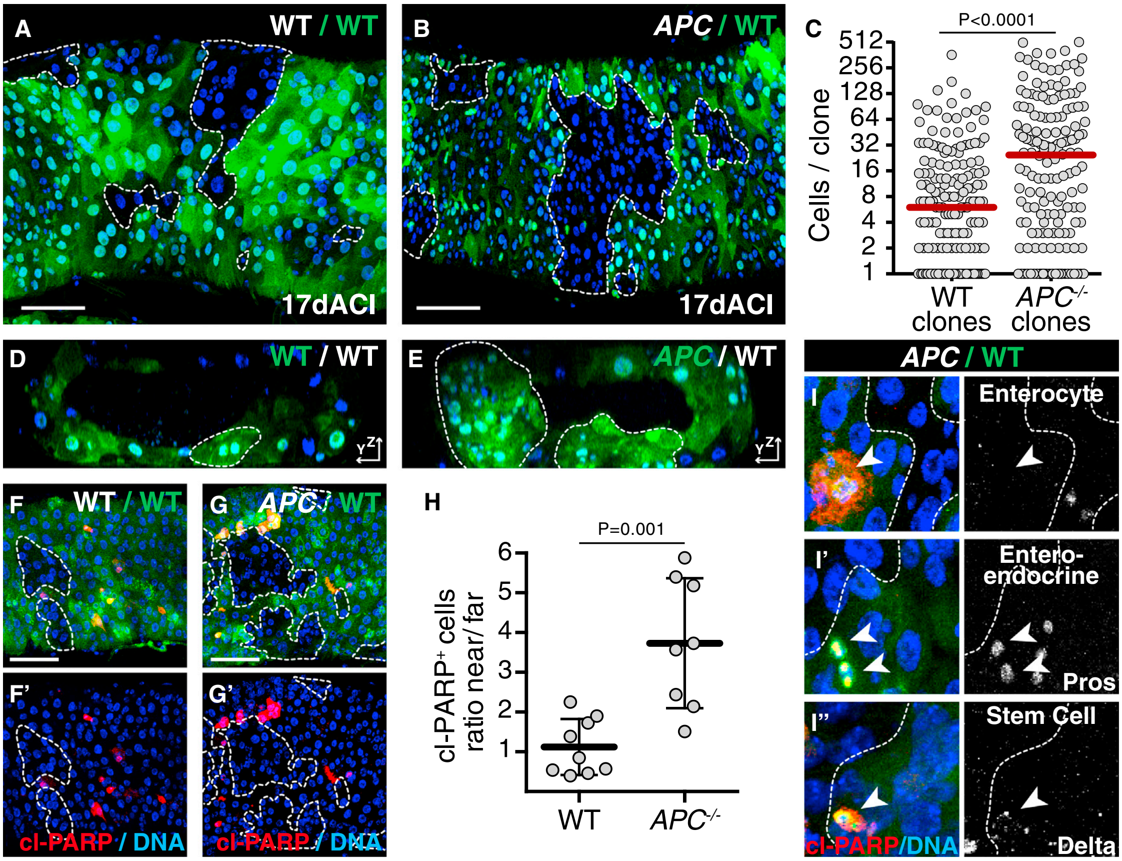

Figure 1. *APC*$^{-/-}$ Adenomas Eliminate Surrounding Cells

(A–E) Posterior midguts harboring control (A and D) or *APC*$^{-/-}$ (B and E) clones, marked by the absence of GFP (A and B) or by 2×GFP (D and E). (A) and (B) show a maximum intensity projection of multiple z sections in x/y. (D) and (E) show a reconstruction of all z sections in y/z. The graph in (C) displays the distribution of clone sizes (left, n = 176 clones; right, n = 164 clones).

(F–I″) Analysis of cell death in posterior midguts harboring control (F and F′) or *APC*$^{-/-}$ (G, G′, and I–I″) clones marked by the absence of hPARP-Venus. Immunostaining for cleaved hPARP (red) marks apoptotic cells. The graph in (H) displays the ratio of cleaved-hPARP-positive cells at clone borders (near) normalized to the rest of the tissue (far). Each dot represents one gut, and the black bar indicates the average ratio (±SD; n = 8 guts per condition). Apoptotic cells around *APC*$^{-/-}$ adenomas are enterocytes (identified by a polyploid nucleus; I), anti-Prospero-positive enteroendocrine cells (I′) or anti-Delta-positive intestinal stem cells (I″); arrowheads point to apoptotic cells.

Throughout the figures, colored lettering describes fluorescent-protein-positive and white lettering fluorescent-protein negative tissue, and dashed lines indicate clone borders. Unless stated otherwise, in the graphs each dot represents one clone, red bars indicate median clone sizes, and p values are displayed above graphs (Mann-Whitney test). Detailed genotypes are listed in the Supplemental Experimental Procedures. Scale bars represent 50 μm.

for tumor growth, tumor-induced cell competition acts as a key driver of tumorigenesis in this tissue, providing a novel angle to counter tumor expansion.

RESULTS

APC$^{-/-}$ Adenomas Induce Apoptosis in Surrounding Cells

To investigate whether cell competition takes place at sites of pre-cancerous lesions, we used mutations in the *Adenomatous polyposis coli* (*Apc*) genes, which cause hyper activation of the Wnt pathway and induce hyperplasia and benign tumor formations (hereafter referred to as adenomas) in the adult *Drosophila* midgut [19, 20]. We focused on Wnt-induced adenomas because we previously showed that in developing tissues, cells with increased Wnt signaling can adopt a supercompetitor phenotype and cause elimination of normal cells [23]. In addition, the mechanisms driving *APC*$^{-/-}$ hyperplasia in the fly show important similarities with *APC*$^{-/-}$ intestinal adenoma growth in mammals (e.g., activation of the oncogene *myc* in *APC*$^{-/-}$ cells and dependence on Myc activity for adenoma growth [24]), making our study potentially relevant to the onset of this pathology.

To generate *APC*$^{-/-}$ intestinal adenomas, we introduced ISCs mutant for *APC1* and *APC2* (hereafter referred to as *APC*$^{-/-}$) in the adult fly posterior midgut by flippase (FLP)-mediated mitotic recombination (Figure S1A). Clones derived from these cells were significantly bigger than control wild-type clones of similar age (Figures 1A–1C) and formed multi-layered structures bulging in the lumen of the gut (Figures 1D and 1E), as previously described [19, 20]. This distorted morphology is visible 10 days after clone induction (ACI; data not shown), but is more prominent at later stages. To address whether these adenomas induce

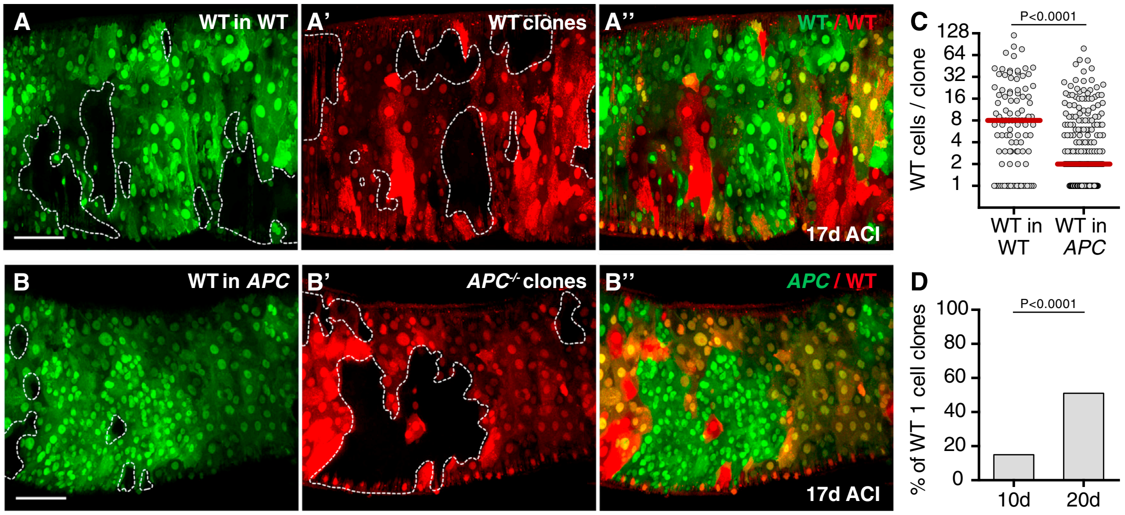

Figure 2. *APC*$^{-/-}$ -Induced Cell Competition Causes Attrition of Healthy Tissue

Posterior midguts harboring simultaneously induced GFP-negative WT (A, A′′, B, and B′′) and RFP-negative WT (A′ and A′′) or *APC*$^{-/-}$ (B′ and B′′) clones. The graph in (C) displays the distribution of WT clone sizes (left, n = 106 clones; right, n = 227 clones). The graph in (D) shows the percentage of one-cell clones across a whole population of WT clones in guts containing *APC*$^{-/-}$ clones dissected 10 or 20 days ACI (left, n = 179 clones; right, n = 74 clones; Fisher's exact test). See also Figure S2.

cell competition, we then looked at the incidence of death in cells surrounding these clones. Using cleavage of PARP as a readout for caspase activation, we observed apoptotic cells in both control guts and guts containing *APC*$^{-/-}$ cells (Figures 1F–1G′). However, although apoptotic cells were randomly distributed in control epithelia (Figures 1F, 1F′, and 1H, left graph), we found a 4-fold enrichment in apoptotic cells around *APC*$^{-/-}$ adenomas (Figures 1G, 1G′, and 1H, right graph). Increased apoptosis was observed both among the differentiated cell types, i.e., enterocytes (recognized by their large polyploid nuclei; Figure 1I) and enteroendocrine cells (marked by expression of Prospero; Figure 1I′), and among ISCs (marked by expression on Delta; Figure 1I′′). Overall, we conclude that growing *APC*$^{-/-}$ adenomas induce elimination of nearby cells by apoptosis.

APC$^{-/-}$-Induced Cell Competition Causes Attrition of Healthy Tissue

The increased elimination of cells surrounding *APC*$^{-/-}$ adenomas urged us to further examine the behavior of the host tissue in proximity of adenomas. By labeling the *APC* mutant and the wild-type chromosomes with different fluorescent markers, we could lineage trace simultaneously induced clones of cells originating either from *APC*$^{-/-}$ (RFP-negative) or from wild-type (GFP-negative) stem cells (Figure S1B). Interestingly, we found that wild-type clones were dramatically smaller when grown in midguts containing *APC*$^{-/-}$ adenomas (Figures 2B and 2C, right graph) than genetically identical control clones grown in wild-type epithelia (Figures 2A and 2C, left graph), with a median clone size of only ~25% of their expected size. In addition, we found that the number of wild-type clones per gut drops drastically over time (Figure S2A), with the majority of residual clones made by one cell only at 20 days ACI (Figure 2D), indicating accelerated clone extinction. Indeed, wild-type clones in control guts showed a much lower incidence of one-cell clones (Figure S2B). Altogether, these data indicate that *APC*$^{-/-}$ adenomas engage in cell competition with surrounding wild-type cells and, by acting as supercompetitors, cause attrition of the host tissue.

Cell Competition Drives Tumor Growth

Loss of healthy cells in a tumor-bearing environment is detrimental to organ function and compromises health [25]. Therefore, we next asked whether we could protect wild-type tissue from elimination induced by *APC*$^{-/-}$ adenomas by expressing inhibitors of apoptosis. Using the GeneSwitch system, which allows RU486 (mifepristone)-inducible Gal4-driven expression, we expressed the *Drosophila* Inhibitor of Apoptosis 1 (DIAP1) or baculovirus protein p35 directly after clone induction, across the posterior midgut in both progenitor cells and enterocytes [22, 26]. Remarkably, we found that inhibition of apoptosis by DIAP1 (Figures 3A–3C) or p35 (Figures S3A–S3C) expression was sufficient to fully restore growth of wild-type clones (Figure 3C; compare also to control clones in Figure 1C, left graph; p = 0.5694). This indicates that apoptotic induction alone can account entirely for host tissue attrition during cell competition.

Strikingly, the growth of *APC*$^{-/-}$ adenomas was drastically reduced in guts in which the loss of neighboring tissue had been prevented (Figures 3A′, 3B′, 3D, S3A′, S3B′, and S3D). In fact, the size of *APC*$^{-/-}$ clones was statistically indistinguishable from that of wild-type clones within the same guts (compare the right graphs in Figures 3C and 3D; p = 0.4211). This was not an indirect effect of inhibition of turnover, since wild-type clone growth was instead rescued in these same guts (Figures 3A–3C and S3A–S3C). In addition, DIAP1 or p35 expression did not affect the behavior of control clones in control guts (Figures S3E and S3F). Two complimentary experiments confirmed that

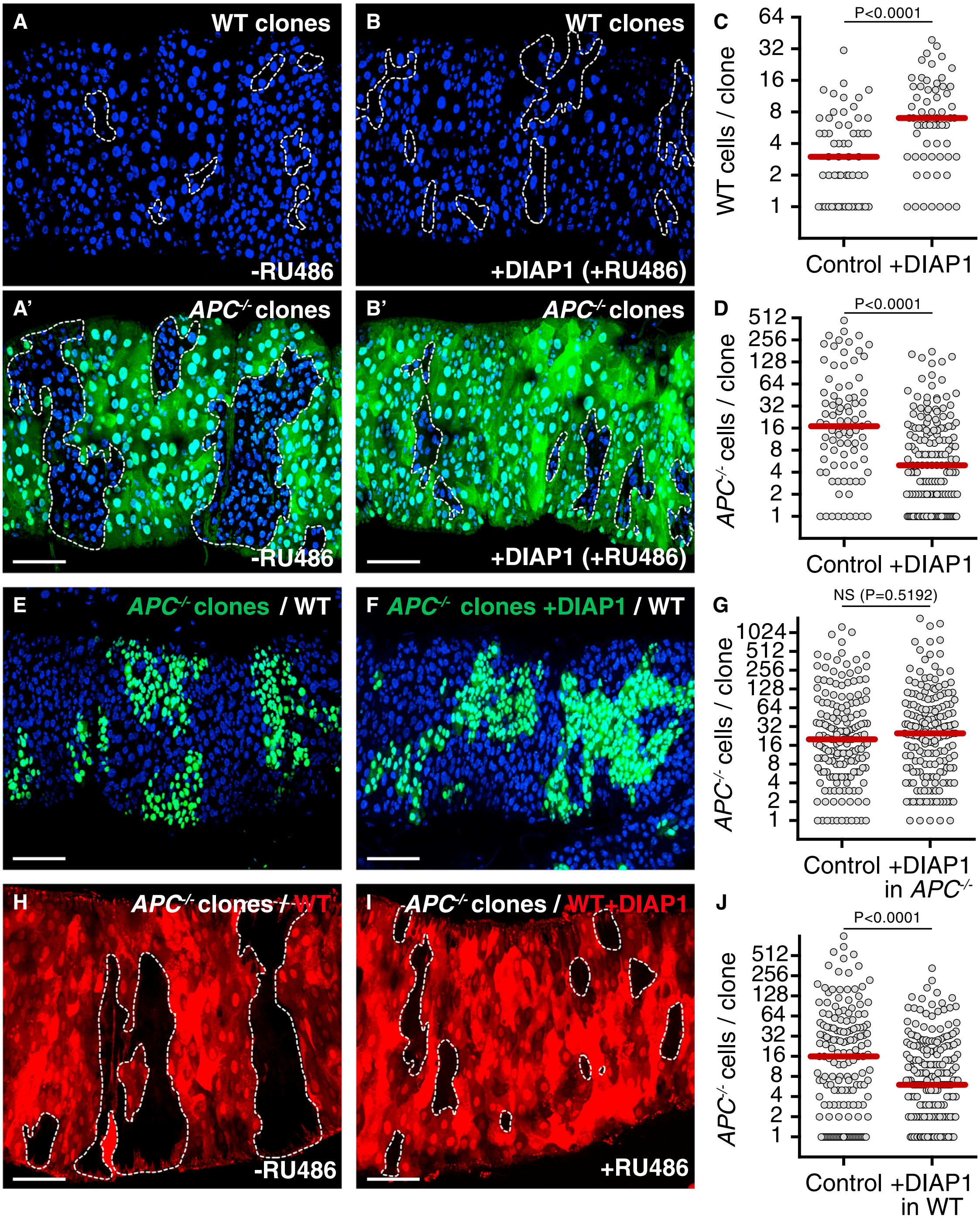

Figure 3. Cell Competition Fuels Tumor Growth

(A–D) Posterior midguts harboring WT clones (A and B) and $APC^{-/-}$ clones, (A′ and B′). Clones are marked by 2×GFP (WT) or by the absence of GFP ($APC^{-/-}$). In (B) and (B′), cell death was blocked by inducible expression of DIAP1 (+DIAP1; 40 μM RU486). Control guts (A and A′) are of the same genotype as in (B) but were treated with carrier only (−RU486). The graphs in (C) and (D) display the distribution of WT (C; left, n = 59 clones; right, n = 63 clones) or $APC^{-/-}$ (D; left, n = 87 clones; right, n = 161 clones) clone sizes.

(E–G) Posterior midguts harboring $APC^{-/-}$ clones marked by expression of GFP, with (F; +DIAP1) or without (E; control) additional expression of DIAP1. The graph in (G) displays the distribution of $APC^{-/-}$ clone sizes with (right) or without (left) DIAP1 expression (left, n = 172 clones; right, n = 187 clones).

(H–J) Posterior midguts harboring $APC^{-/-}$ clones marked by the absence of RFP. In (I), cell death was blocked in host cells by expression of DIAP1 (+DIAP1; 40 μM RU486). Control guts (H) are of the same genotype as in (I) but were treated with carrier only (−RU486). The graph in (J) displays the distribution of $APC^{-/-}$ clone sizes with (right) or without (left) DIAP1 expression in host cells (left, n = 160 clones; right, n = 218 clones).

See also Figure S3.

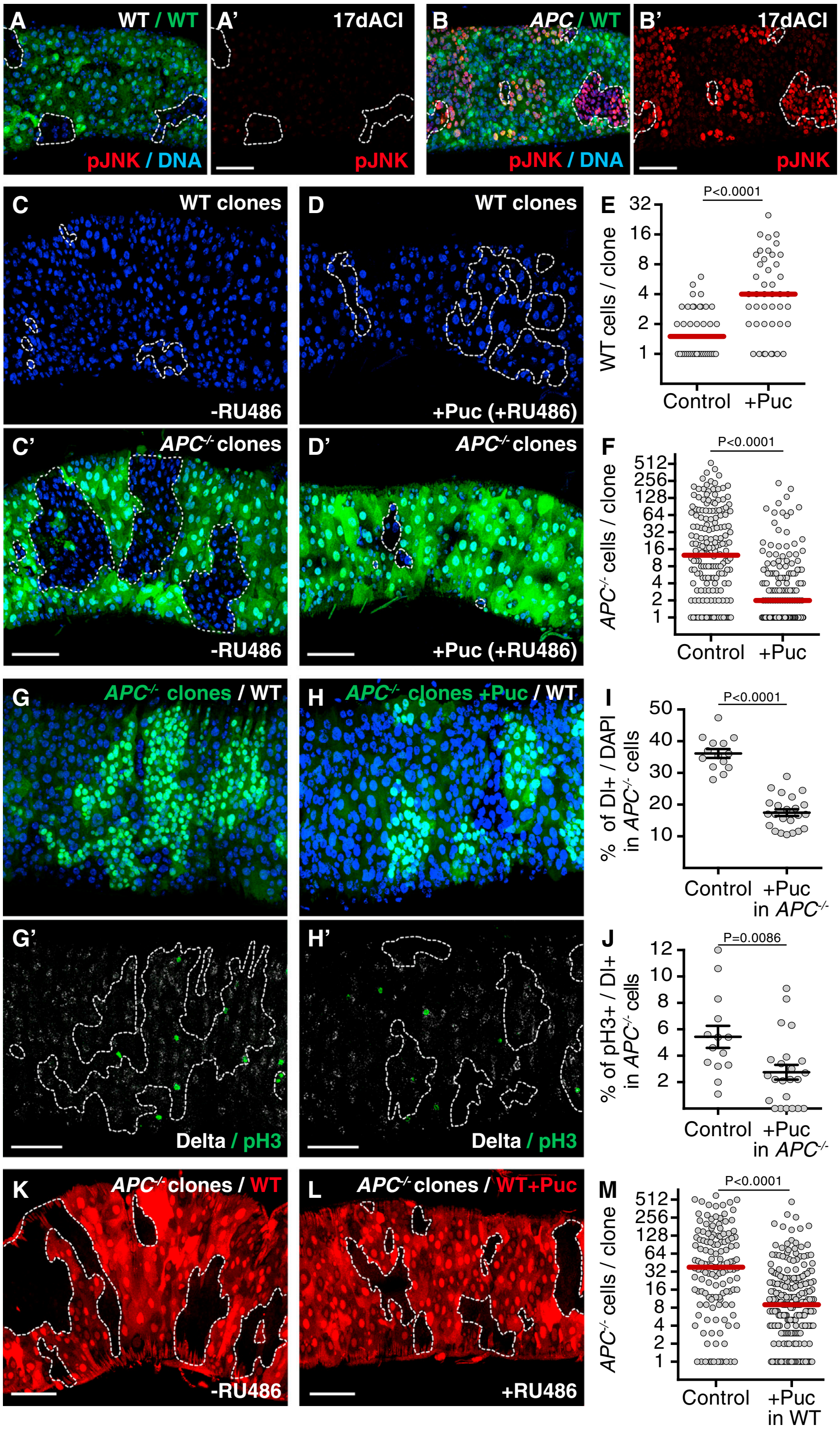

Figure 4. JNK Signaling Boosts $APC^{-/-}$ Adenoma Growth

(A–B′) Posterior midguts stained with anti-phospho (active) JNK (pJNK, red) containing WT (A and A′) or $APC^{-/-}$ clones (B and B′) marked by the absence of GFP.

(C–F) Posterior midguts harboring WT clones, marked by 2×GFP (C′ and D′ and outlined in C and D), and $APC^{-/-}$ clones, marked by the absence of GFP (C′ and D′). JNK signaling was blocked by inducible expression of Puckered (+Puc; 200 μM RU486; D and D′). Control guts (C and C′) are of the same genotype as in (D) but were treated with carrier only (−RU486). The graphs in (E) and (F) display the distribution of clone sizes for WT clones (E; left, n = 38 clones; right, n = 44 clones) and $APC^{-/-}$ clones (F; left, n = 184 clones; right, n = 161 clones).

(G–J) Posterior midguts harboring $APC^{-/-}$ clones marked by expression of GFP with (H) or without (G) additional expression of Puckered within the clones and stained with anti-phospho H3 to mark mitotic cells (green) and anti-Delta to mark ISCs (white). The graphs in (I) and (J) display the percentage of $APC^{-/-}$ Delta-positive stem cells (I) or the percentage of mitotic $APC^{-/-}$ Delta-positive stem cells per gut (J). Each dot represents one gut, and the black bar indicates the average (±SEM; left, n = 14 guts; right, n = 23 guts; t test).

(K–M) Posterior midguts harboring $APC^{-/-}$ clones marked by the absence of RFP. In (L), JNK signaling was blocked in host cells by inducible expression of Puckered (+Puc; 40 μM RU486). Control guts (K) are of the same genotype as in (L) but were treated with carrier only (−RU486). The graph in (M) displays the distribution of $APC^{-/-}$ clone sizes (left, n = 137 clones; right, n = 227 clones).

See also Figure S4.

this effect is due to inhibition of apoptosis specifically in the host tissue. First, expression of DIAP1 or p35 only in $APC^{-/-}$ cells did not affect their clone size (Figures 3E–3G and S3G–S3H), ruling out an autonomous effect. Second, conditional inhibition of apoptosis exclusively in the host tissue (see Figures S1C and S1D for genetic setup) reduced growth of $APC^{-/-}$ adenomas to a similar extent as inhibition throughout the epithelium (Figures 3H–3J; compare the right graphs in Figures 3D and 3J; p = 0.7212). Collectively, these data demonstrate that tumor-host cell competition is essential to drive the growth of $APC^{-/-}$ adenomas in the *Drosophila* adult midgut.

JNK Signaling Boosts $APC^{-/-}$ Adenoma Growth Autonomously and via Cell Competition

We next wondered which pathways are involved in $APC^{-/-}$ adenoma expansion. The Jun N-terminal kinase (JNK) pathway plays a fundamental role in modulating both cell proliferation and cell death in many tissues, including the fly intestine [27–29], and has been shown to be required for loser cell elimination in several types of cell competition [30, 31]. Using a phospho-specific antibody that recognizes an activated form of JNK, we observed high JNK activation specifically in guts that contain $APC^{-/-}$ adenomas (Figures 4B, 4B′, S4A, and S4A′), but not in control wild-type (Figures 4A and 4A′) or heterozygous $APC^{-/+}$ (Figure S4B) guts.

Hyper-activation of JNK was prominent both inside $APC^{-/-}$ adenomas and in surrounding tissue (Figure 4B). This was not an effect of tissue aging [28] because increased pJNK signal could be observed as early as 5 days ACI (Figures S4C and S4D). Importantly, pJNK staining was still present within $APC^{-/-}$ clones in guts in which competition had been blocked by apoptosis inhibition (Figures S4E and S4F, arrowhead); however, its levels were reduced in small $APC^{-/-}$ clones (Figure S4F), indicating that clone size is important for JNK activation.

We next tested the relevance of JNK activation to $APC^{-/-}$ adenoma growth and cell competition. Interestingly, inhibition of the pathway throughout the gut epithelium, by GeneSwitch-induced expression of the JNK inhibitor Puckered (Puc), rescued wild-type clone size (Figures 4C, 4D, and 4E). Notably, the growth of $APC^{-/-}$ adenomas was severely reduced under these conditions (Figures 4C′, 4D′, and 4F). Since JNK can have a pro-proliferative effect, we then asked whether the reduction in $APC^{-/-}$ clone growth was due to a cell-autonomous effect. Importantly, we found that JNK inhibition in $APC^{-/-}$ cells, by expression of Puc or a dominant-negative version of JNK (JNK^{DN}) caused a marked reduction in $APC^{-/-}$ clone size (Figures 4G–4H and S4G–S4I). This was accompanied by a reduction of the proliferation rate and of the proportion of ISCs in $APC^{-/-}$ clones (Figures 4G–4J), both of which have been shown to be increased in $APC^{-/-}$ tumors [19, 20, 24]. This indicates that JNK signaling is required both for proliferation and for stem cell fate maintenance in $APC^{-/-}$ cells. Note that dependence on JNK activity for clonal expansion is not a general feature of ISCs, as JNK signaling inhibition has no effect on the colonization of wild-type cells in control guts [22]. Next, to dissect the role of JNK signaling in cell competition, we inhibited the pathway in non-tumor cells only. Importantly, inducible expression of either Puc or JNK^{DN} specifically in the host tissue severely reduced growth of $APC^{-/-}$ adenomas (Figures 4K–4M and S4J–S4L). Together, these data indicate that JNK signaling has a dual function: it is required in $APC^{-/-}$ cells to promote their growth and in loser cells for their elimination by cell competition.

It has been shown that, in the fly intestine, expression of the secreted JAK/STAT cytokine Unpaired-3 (Upd-3) can be activated by JNK signaling upon stress or injury [22, 27, 29]. Furthermore, the growth of $APC^{-/-}$ clones has been reported to be JAK/STAT dependent [24]. However, inhibition of JNK signaling in the host tissue by expression of JNK^{DN} did not abrogate elevation of JAK/STAT signaling in $APC^{-/-}$ adenomas (Figures S4M and S4N). Thus, inhibition of JNK signaling in neighboring cells blocks cell competition in a JAK/STAT-independent manner.

Tumor Growth Is Required for Cell Competition

By monitoring competing clones at 10 and 20 days ACI, we observed that wild-type clones initially grew (Figures 5A and 5C, left graph) and subsequently shrunk (Figures 5B and 5C, right graph). This coincided with an increase in $APC^{-/-}$ clone size (Figures 5A′, 5B′, and 5D), suggesting that $APC^{-/-}$ clones need to attain a critical size to compete efficiently. Indeed, blocking $APC^{-/-}$ clone growth by inhibiting JNK signaling or silencing of Myc [24] was sufficient to rescue wild-type clone size (Figures 5E–5J). We found that guts containing $APC^{-/-}$ clones with an average size of $\sim$30 cells were able to outcompete wild-type clones (Figure S5A; compare to control size in Figures 2A and 2C, left graph), indicating that this is a sufficient size for $APC^{-/-}$-induced competition. Notably, although *myc* is upregulated in *Drosophila* $APC^{-/-}$ intestinal adenomas and required for their overgrowth [24], we found that increasing Myc expression in host cells did not rescue their outcompetition (Figure S5B) or inhibit $APC^{-/-}$ adenoma growth (Figures 5K–5M). This indicates that, like in developing epithelia [23], differences in Myc levels are not required for $APC^{-/-}$-induced cell competition in the intestine.

Relative Differences in Hippo Activity Determine the Cell Competition Potential of $APC^{-/-}$ Cells

The Hippo pathway plays an important role in growth control and can inhibit proliferation and promote apoptosis via inhibitory phosphorylation of the downstream transcriptional co-activators YAP and TAZ (Yorkie [Yki] in *Drosophila*) [32]. Given that Hippo signaling has been implicated in cell competition in developing tissues [33–35] and that Wnt signaling induces YAP/TAZ activation in mammals [36, 37], we investigated whether Yki is active in $APC^{-/-}$ adenomas and whether it plays a role in cell competition. First, we observed that activity of the microRNA and Yki target gene *bantam* was high (Bantam-GFP levels were low) in some $APC^{-/-}$ clones (Figures S6A and S6B). Second, *diap1*-LacZ, another reporter of Yki activity, was consistently upregulated in $APC^{-/-}$ adenomas (Figures 6A–6A″). Interestingly, *diap1*-LacZ upregulation was seen predominantly in small cells (Figures 6A–6A″, compare inset 1 to inset 2) and was observed throughout $APC^{-/-}$ clones and not just at clone borders, where cell competition takes place, suggesting that upregulation of Yki activity is autonomous to $APC^{-/-}$ cells and not a consequence of cell competition. Consistent with this, inhibiting cell competition by blocking apoptosis (Figures 6B–6B″) or JNK signaling (Figures S6C and S6D) in the host tissue did not affect the ectopic activation of *diap1*-LacZ, despite the severe reduction in clone size.

To test the involvement of Hippo signaling in cell competition, we aimed to level differences in Yki activity between $APC^{-/-}$ clones and their surrounding host tissue. Thus, we removed one functional copy of the upstream inhibitory kinase Hippo ($hpo^{42\text{-}47/+}$) or its upstream activator Expanded ($ex^{ex1/+}$), with the aim of marginally decreasing pathway activity across the gut. Importantly, we found that halving the *hpo* or *ex* gene dosage fully rescued the growth ability of otherwise wild-type clones (Figures 6C–6F). This was not a consequence of a general hyper-proliferative response to *hpo* or *ex* heterozygosity, as it did not have any effect on clonal growth in otherwise wild-type guts (Figures S6E–S6H). Thus, imperceptibly tweaking Hippo activity is sufficient to abrogate $APC^{-/-}$-induced cell competition in this tissue. Strikingly, the growth of $APC^{-/-}$ adenomas was severely reduced in $hpo^{-/+}$ or $ex^{-/+}$ heterozygous backgrounds (Figures 6C′–6E′ and 6G). This is extremely unexpected, because removing one copy of a tumor suppressor should instead promote the proliferative potential of tissues. In contrast, the median size of these $APC^{-/-}$ clones reverted to that of wild-type clones in the same tissue (for $hpo^{-/+}$, compare Figures 6D and 6D′ and the middle graphs in Figures 6F and 6G; $p = 0.3744$; for $ex^{-/+}$, compare Figures 6E and 6E′ and the right graphs in Figures 6F and 6G; $p = 0.7621$). Importantly, this was not caused by a detrimental effect of Yki activity on $APC^{-/-}$ adenomas, because autonomous overexpression of Yki in $APC^{-/-}$ cells did not inhibit their growth (Figures S6I–S6K). Notably, the suppression of

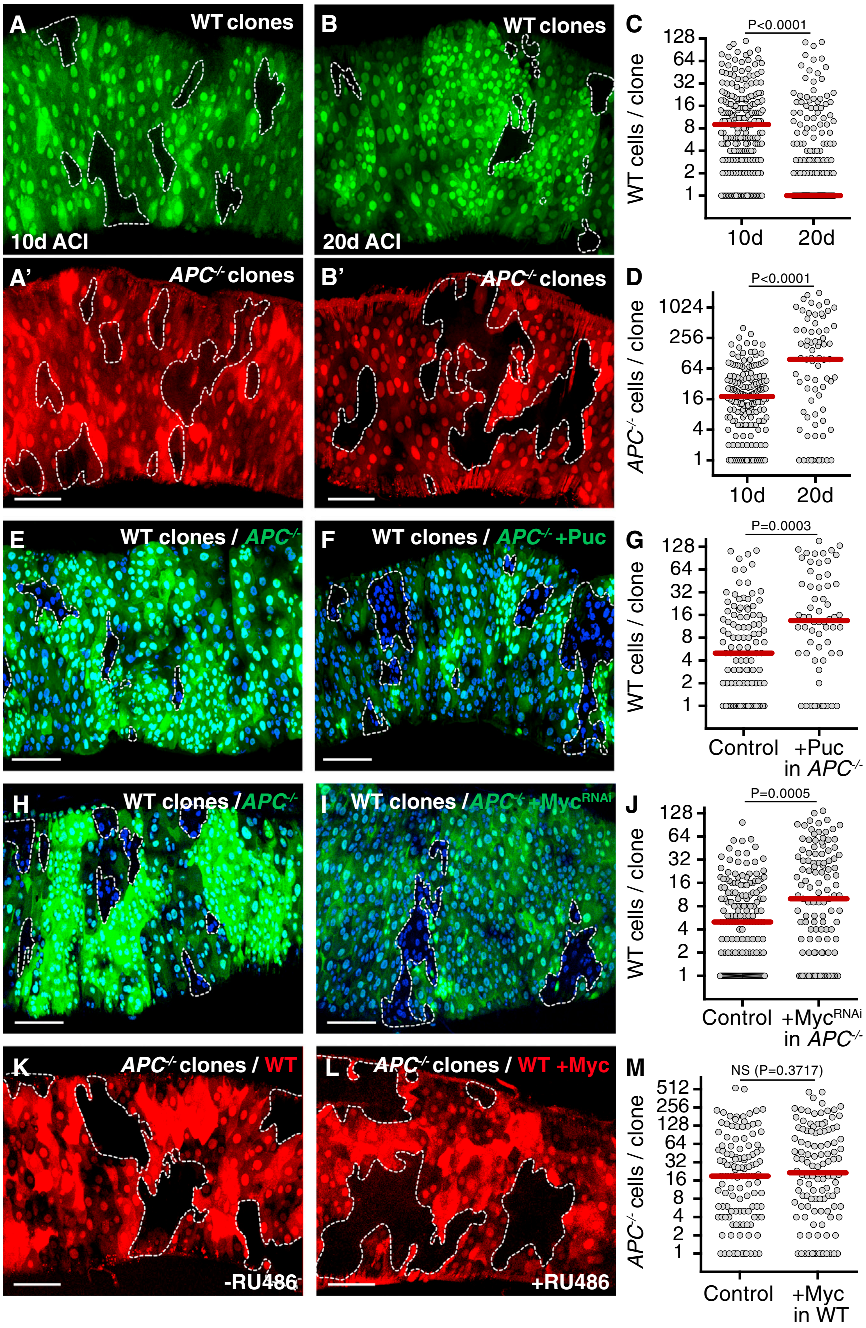

Figure 5. Tumor Growth Is Required for Cell Competition

(A–D) Posterior midguts harboring simultaneously induced GFP-negative WT (A and B) and RFP-negative *APC*$^{-/-}$ (A′ and B′) clones, dissected 10 days (A) or 20 days (B) ACI. The graphs in (C) and (D) display the distribution of clone sizes for WT clones (C; left, n = 179 clones; right, n = 74 clones) or *APC*$^{-/-}$ clones (D; left, n = 231 clones; right, n = 186 clones).

(E–J) WT clones, marked by the absence of GFP, in posterior midguts harboring control *APC*$^{-/-}$ clones (E and H) or *APC*$^{-/-}$ clones expressing Puckered (F) or Myc^{RNAi} (I) specifically within the clone. The graphs in (G) and (J) display the distribution of WT clone sizes (G, left, n = 112 clones; G, right, n = 62 clones; J, left, n = 164 clones; J, right, n = 118 clones).

(K–M) Posterior midguts harboring *APC*$^{-/-}$ clones marked by the absence of RFP. Myc was inducibly expressed in host cells (L; +Myc; 40 μM RU486). Control guts (K) are of the same genotype as in (L) but were treated with carrier only. The graph in (M) displays the distribution of *APC*$^{-/-}$ clone sizes (left, n = 119 clones; right, n = 114 clones).

See also Figure S5.

APC$^{-/-}$ adenoma growth by *hpo* heterozygosity was not due to a reduction in JNK (data not shown) or JAK-STAT (Figures S6L and S6M) signaling. Altogether, these results show that Yki signaling is activated in *APC*$^{-/-}$ adenomas and plays a role in *APC*$^{-/-}$-induced cell competition in the intestine and that differences in Hippo signaling, rather than absolute Hippo activity, determine the cell competition potential of *APC*$^{-/-}$ adenomas.

DISCUSSION

It has been well over a decade since the first reports of a connection between cancer-related genes and cell competition [8, 9]. These and a panoply of subsequent studies led to the long-standing hypothesis that cell competition contributes to cancer formation [10, 11]. Here we have investigated this directly, by exploiting the recent establishment of the adult *Drosophila* intestine as a model system to study adult tissue homeostasis and tumor formation [15, 18]. Our work shows that Wnt-induced intestinal adenomas directly compete with the host tissue. Importantly, we find that cell competition is an essential driver of tumor growth. Indeed, inhibiting cell competition suppresses over-proliferation in *APC*$^{-/-}$ cells, effectively blocking tumor formation (Figure 7). Importantly, this finding demonstrates that the growth of cells with a mutation considered to be a major driver of colon cancer is not uncontrolled and that the cellular environment plays a deterministic role in the behavior of those cells. In this light, some previously reported observations might, at least in part, be explained by cell competition. For example, it has been reported that not all micro-metastases have the potential to immediately grow into secondary tumors [38], a phenomenon called cancer dormancy [38]. Based on our findings, we speculate that the interaction of such micro-metastasis with their environment, through cell competition, could play a deterministic role in their ability to grow or not. Consistent with this hypothesis, it has been shown that in developing *Drosophila* tissues cells with mutations in some tumor suppressor genes (e.g., *lgl* and *scribble*) can be eliminated by wild-type cells [39]. It is only through

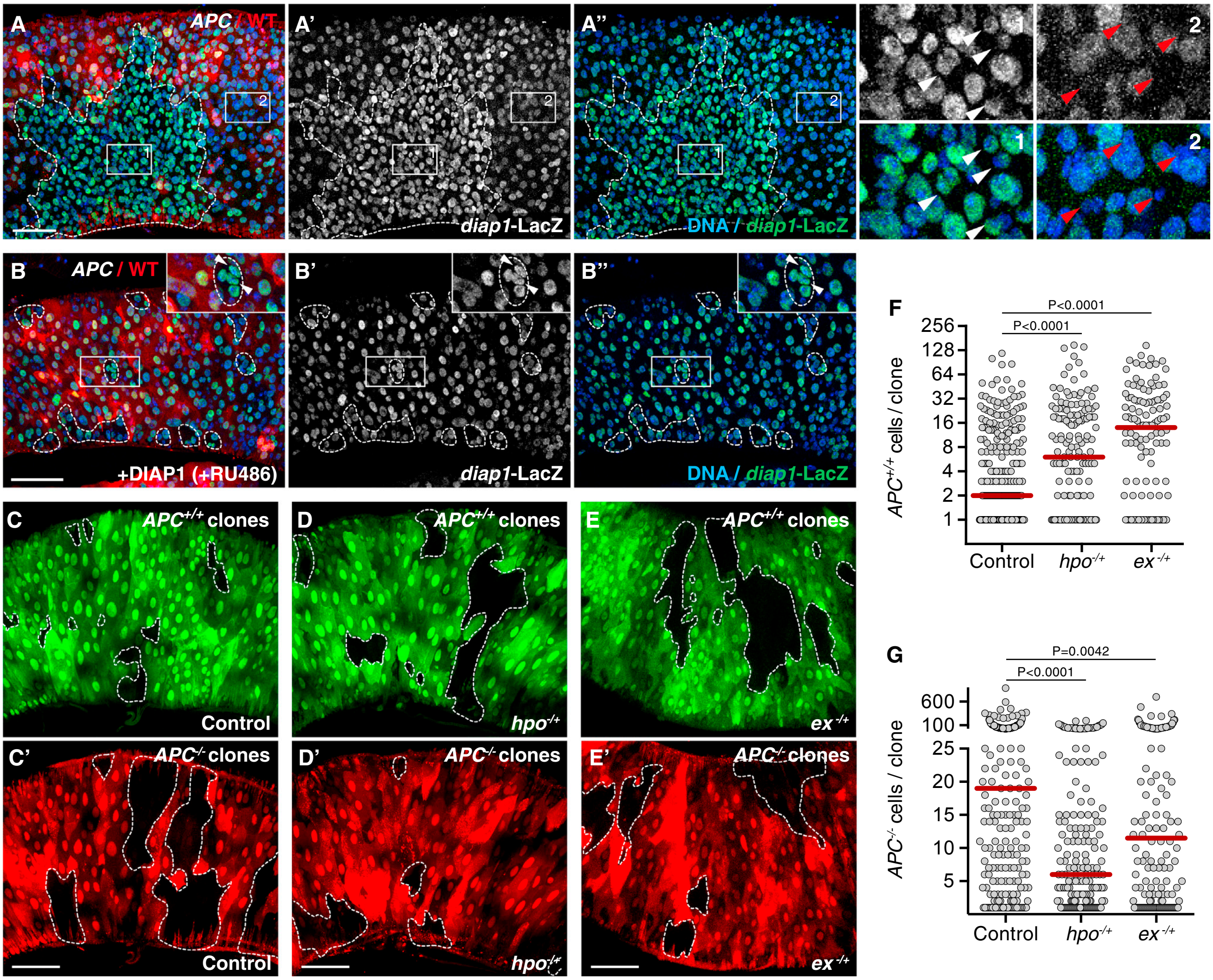

Figure 6. Differences in Hippo Activity Determine Cell Competition Potential of *APC*$^{-/-}$ Cells

(A–B$''$) Posterior midguts with *APC*$^{-/-}$ clones, marked by the absence of RFP 17 days (B) or 20 days (A) ACI. Yki activity was detected by expression of *diap1*-LacZ (white, A$'$ and B$'$; green, A, A$''$, B, and B$''$). The magnifications in (A) display regions inside (region 1) and outside (region 2) *APC*$^{-/-}$ clones, and arrowheads point at small *APC*$^{-/-}$ mutant (white arrowheads) or small WT (red arrowheads) cells. Cell death was blocked in (B) by inducible expression of DIAP1 (40 μM RU486). The magnifications display regions containing *APC*$^{-/-}$ clones, and arrowheads point at small *APC*$^{-/-}$ mutant cells.

(C–G) Guts containing simultaneously induced GFP-negative WT (C, D, and E) and RFP-negative *APC*$^{-/-}$ (C$'$, D$'$, and E$'$) clones in control (C and C$'$), *hpo*$^{-/+}$ (D and D$'$), or *ex*$^{-/+}$ (E and E$'$) posterior midguts. The graphs in (F) and (G) display the distribution of *APC*$^{+/+}$ (F; left, n = 304 clones; middle, n = 155 clones; right, n = 120 clones) or *APC*$^{-/-}$ (G; left, n = 237 clones; middle, n = 183 clones; right, n = 158 clones) clone sizes from control (left), *hpo*$^{-/+}$ (middle), or *ex*$^{-/+}$ (right) guts. See also Figure S6.

acquisition of additional mutations (similarly to "second hit" mutations during tumorigenesis) that those cells overcome the tumor-suppressive environment of the host and overgrow [31, 40]. Furthermore, it has recently been found that naturally occurring cell competition in the thymus protects mice from developing leukemia [41], lending further support to this notion.

Recently, it has been shown that some mutations involved in human colon cancer can give a competitive advantage to cells in the mouse gut. Specifically, oncogenic mutations in K-Ras [42] or APC [43] endow stem cells with a competitive advantage, which increases their chances of colonization. On the basis of clone population dynamics, those studies have proposed that cell-autonomous differences in cell proliferation or cell survival rates among wild-type and oncogenically mutated cells account for their colonization bias. Here we have taken a different approach, whereby at the same time as scoring adenoma growth we monitored and manipulated the cell survival probability of the host tissue. This has allowed us to uncover cell interactions among tumor and host cells that cause induction of cell death in surrounding normal tissue, a feature that we demonstrate to be essential to enable adenoma growth. In light of our findings, we suggest that a similar process may contribute to the colonization bias observed in the mouse intestine [42, 43].

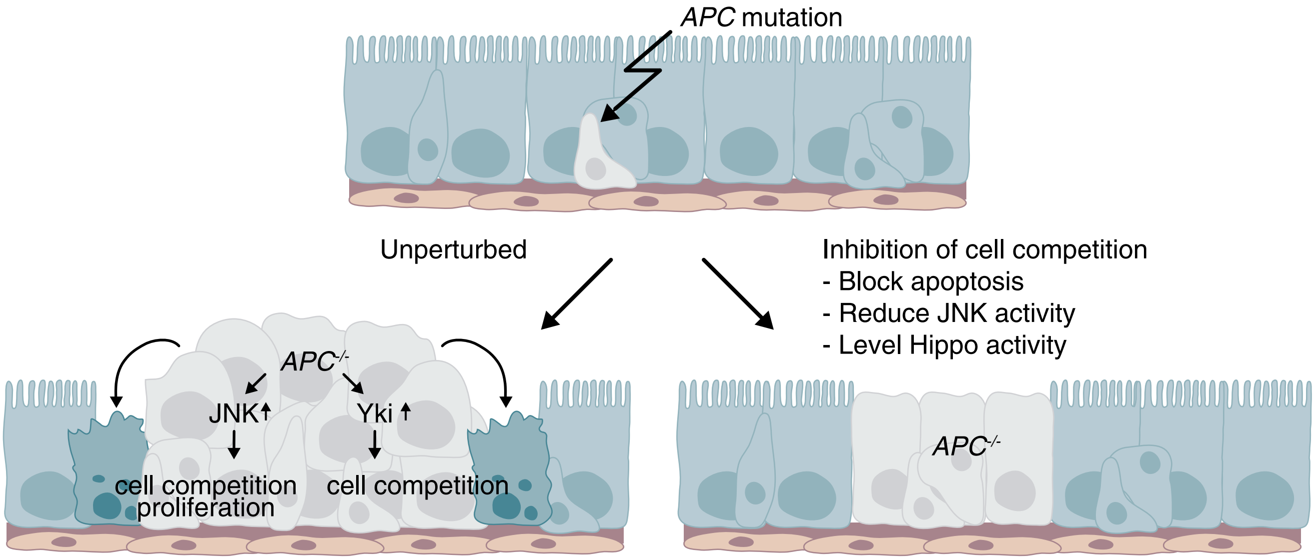

Figure 7. Cell Competition Promotes Tumor Growth in *Drosophila*
Schematic model depicting how cell competition affects $APC^{-/-}$ adenoma growth. Growing $APC^{-/-}$ adenomas in the adult *Drosophila* posterior midgut kill surrounding cells and cause host tissue attrition. JNK signaling activation in $APC^{-/-}$ cells is required for their growth, whereas non-autonomous JNK activation in the host tissue is required for cell competition. $APC^{-/-}$ cells also display higher Yki activity than host cells and this is required for cell competition. Inhibition of cell competition prevents adenoma growth, indicating that cell competition is an essential driver of tumor growth in this tissue.

We further show that growing $APC^{-/-}$ adenomas cause accelerated extinction of wild-type competing clones, resulting in attrition of surrounding tissue. This is remarkable, considering that it has been shown that *APC* mutations induce a cytokine-rich pro-proliferative environment in and around adenomas, which should instead promote growth [24]. This indicates that host tissues recede at sites of tumor growth, a process that is not only disadvantageous because it enhances tumorigenesis, but is also detrimental to organ performance. Since interfering with tumor growth inhibits competition and, vice versa, inhibiting cell competition blocks tumor growth, we propose that both events occur simultaneously and enhance one another in a feed-forward loop. Our finding that apoptosis inhibition allows the host tissue to contain growing adenomas could have important implications for cancer therapy, as it could provide a strategy to prevent or delay a lethal aspect of cancer, namely organ failure [25]. It further suggests that apoptosis inhibitors might constitute an unexplored arsenal in combination therapies against cancer. This is a radical suggestion, given that many anti-cancer chemo- and radiotherapies are, on the contrary, based on the use of wide-spectrum cell death inducers.

Our work identifies a new role for Yki activity in tumor growth. In particular, we show that $APC^{-/-}$ tumors display increased Yki activity, consistent with previous findings [36, 37]. Since YAP/TAZ and Yki are oncogenes, it is paradoxical that halving the gene dosage of *hpo* or *ex*, both of which are Yki inhibitors and recognized/putative tumor suppressors, should inhibit adenoma growth. This points at a new unappreciated role of Hippo signaling, which provides $APC^{-/-}$ adenomas with the ability to compete. Importantly, it further highlights that relative rather than absolute differences in Hippo activity are important for tumor growth. A *hpo* or *ex* heterozygous background (but, interestingly, not *yki* heterozygosity; Figures S6O and S6P) most likely limits the ability of Hippo to inhibit Yki in the host tissue. We show that although that has no noticeable effect on the behavior of otherwise wild-type cells under normal conditions, it is sufficient to allow them to withstand the competition from $APC^{-/-}$ adenomas. There are several possible mechanistic explanations for this observation. First, the Hippo pathway is an important sensor of cell density [44]. This might be relevant because $APC^{-/-}$ tumors disregard the normal morphology of the midgut epithelium and exhibit higher cell density [20] (Figure 1). Therefore, one could speculate that leveling Yki activity could give surrounding cells a chance to be less sensitive to cell density and thereby prevent cell competition. Alternatively, *hpo* or *ex* heterozygosity may confer some resistance to cell death induction, as one of the targets of Yki is the inhibitor of apoptosis DIAP1 [45]. Lastly, there is evidence that the crosstalk between Hippo and Wnt pathways is bidirectional and that, besides the previously discussed activation of YAP/TAZ by Wnt, the Hippo pathway can also restrict Wnt signaling [46]. Reduced Hippo signaling in surrounding cells could therefore act as a positive feedback to facilitate Wnt activation in these cells.

Finally, our findings also reveal an important role of the JNK pathway in $APC^{-/-}$-driven adenoma formation. As we show, JNK activation in $APC^{-/-}$ cells and in patches of surrounding tissue is important to drive tumor growth. A similar activation of JNK has also recently been observed around, but not inside, intestinal $Notch^{-/-}$ tumors [21]. Interestingly, however, while $Notch^{-/-}$ tumors rely on the niche microenvironment to supply proliferative JAK/STAT ligands, we find that $APC^{-/-}$ tumors, which also require JAK/STAT activation [24], do not depend on a supply from the niche. Both JNK and JAK/STAT pathways are involved in sensing stress, injury, and inflammation and enabling regeneration and repair in the *Drosophila* adult gut [27–29]. This is particularly relevant because there are many

 ©2016 The Authors

reports that inflammation and stress influence tumorigenesis. For example, colitis, induced by dextran sodium sulfate feeding, can strongly promote carcinogenesis in APC^{min} mice [47] and increase the colonization potential of p53 mutant cells [43]. Furthermore, it has been shown that chronic inflammation causes a predisposition for colorectal cancer [48], while treatment with anti-inflammatory drugs decreases this probability [49]. In this regard, we speculate that targeting JNK signaling could provide a particularly effective therapeutic strategy, as it could simultaneously inhibit cancer cell growth and protect host tissue from competition-induced attrition.

Overall, our findings shed light on new potential strategies for cancer treatment. They suggest that the growth of early lesions or micro-metastases could be more effectively prevented by strengthening the surrounding healthy tissue, in addition to focusing on killing the cancer cells themselves, which is the main goal of current treatments.

EXPERIMENTAL PROCEDURES

Drosophila Genetics and Stock Maintenance

Detailed information about the *Drosophila* stocks is given in the Supplemental Experimental Procedures, along with a list of all the experimental genotypes.

Flies were grown at 25°C and fed on standard fly food containing yeast. For experiments using the GeneSwitch system [50], food was supplemented with 40 or 200 μM RU486 (mifepristone; Sigma-Aldrich, M8046) in 80% EtOH or with an equal volume of 80% EtOH as control. Single-stem-cell-derived clones were generated by mitotic recombination using the FLP/FRT (flippase recognition target) system. 1 to 2 days after eclosion, fertilized female flies were heat shocked in a water bath at 37°C for 10 min. Adults were transferred to fresh vials every 2–3 days and were kept at 25°C until dissection at day 17 unless stated otherwise.

Immunostaining

Guts were dissected in PBS and fixed for 20 min at room temperature in PBS containing 3.7% formaldehyde and 0.025% Triton X-100. After several washes in 0.25% Triton X-100/PBS (washing buffer), guts were blocked for 30 min in a solution of 0.1% BSA/0.1% Triton X-100/PBS (blocking buffer). They were then incubated in the appropriate primary antibody diluted in blocking buffer overnight at 4°C. After several washes in washing buffer, guts were incubated for 2 hr at room temperature with the appropriate secondary antibody, followed by several washes in washing buffer. Samples were mounted in Vectashield (Vector Laboratories) on a borosilicate glass slide (no. 1.5, VWR International). A list of the antibodies used is given in the Supplemental Experimental Procedures.

SUPPLEMENTAL INFORMATION

Supplemental Information includes Supplemental Experimental Procedures and six figures and can be found with this article online at http://dx.doi.org/10.1016/j.cub.2015.12.043.

AUTHOR CONTRIBUTIONS

S.J.E.S. and E.P. conceived, designed, and analyzed experiments. S.J.E.S. and G.K. performed experiments, with help from I.K. S.J.E.S. and E.P. wrote the manuscript with help from G.K.

ACKNOWLEDGMENTS

This work was supported by a Cancer Research UK (CRUK) Programme Grant to E.P. and G.K. (A12460); a Royal Society University Research fellowship to E.P. (UF0905080); an EMBO Long-Term Fellowship (ALTF 1476-2012), a NWO Rubicon grant (825.12.027), and a Dutch Cancer Society fellowship (BUIT-2013-5847) to S.J.E.S.; a Wellcome Trust PhD studentship to I.K.; and Core grant funding from the Wellcome Trust Core (092096) and CRUK (C6946/A14492). We thank Rafael E. Carazo Salas and Laura J. Wagstaff for discussions and/or critical reading of the manuscript and our anonymous reviewers for thoughtful and constructive feedback. We thank Emily Dudgeon and the Gurdon Institute Imaging and core facilities for technical assistance and members of the E.P. and St Johnston labs for fruitful input and discussions. We also thank the Bloomington Stock Center (Indiana University) and scientists listed in the Supplemental Experimental Procedures for *Drosophila* strains and the Developmental Studies Hybridoma Bank for antibodies. We apologize to the many researchers whose work could not be cited due to space limitations.

Received: June 19, 2015
Revised: November 2, 2015
Accepted: December 9, 2015
Published: February 4, 2016

REFERENCES

1. Dvorak, H.F. (2015). Tumors: wounds that do not heal-redux. Cancer Immunol. Res. *3*, 1–11.
2. Kalluri, R., and Zeisberg, M. (2006). Fibroblasts in cancer. Nat. Rev. Cancer *6*, 392–401.
3. Kuilman, T., and Peeper, D.S. (2009). Senescence-messaging secretome: SMS-ing cellular stress. Nat. Rev. Cancer *9*, 81–94.
4. Bissell, M.J., and Hines, W.C. (2011). Why don't we get more cancer? A proposed role of the microenvironment in restraining cancer progression. Nat. Med. *17*, 320–329.
5. Mintz, B., and Illmensee, K. (1975). Normal genetically mosaic mice produced from malignant teratocarcinoma cells. Proc. Natl. Acad. Sci. USA *72*, 3585–3589.
6. Pierce, G.B., Pantazis, C.G., Caldwell, J.E., and Wells, R.S. (1982). Specificity of the control of tumor formation by the blastocyst. Cancer Res. *42*, 1082–1087.
7. Stoker, M.G., Shearer, M., and O'Neill, C. (1966). Growth inhibition of polyoma-transformed cells by contact with static normal fibroblasts. J. Cell Sci. *1*, 297–310.
8. de la Cova, C., Abril, M., Bellosta, P., Gallant, P., and Johnston, L.A. (2004). Drosophila myc regulates organ size by inducing cell competition. Cell *117*, 107–116.
9. Moreno, E., and Basler, K. (2004). dMyc transforms cells into super-competitors. Cell *117*, 117–129.
10. Baker, N.E., and Li, W. (2008). Cell competition and its possible relation to cancer. Cancer Res. *68*, 5505–5507.
11. Moreno, E. (2008). Is cell competition relevant to cancer? Nat. Rev. Cancer *8*, 141–147.
12. Morata, G., and Ripoll, P. (1975). Minutes: mutants of Drosophila autonomously affecting cell division rate. Dev. Biol. *42*, 211–221.
13. Vivarelli, S., Wagstaff, L., and Piddini, E. (2012). Cell wars: regulation of cell survival and proliferation by cell competition. Essays Biochem. *53*, 69–82.
14. Vincent, J.-P., Fletcher, A.G., and Baena-Lopez, L.A. (2013). Mechanisms and mechanics of cell competition in epithelia. Nat. Rev. Mol. Cell Biol. *14*, 581–591.
15. Ayyaz, A., and Jasper, H. (2013). Intestinal inflammation and stem cell homeostasis in aging Drosophila melanogaster. Front. Cell. Infect. Microbiol. *3*, 98.
16. Lemaitre, B., and Miguel-Aliaga, I. (2013). The digestive tract of Drosophila melanogaster. Annu. Rev. Genet. *47*, 377–404.
17. Jiang, H., and Edgar, B.A. (2012). Intestinal stem cell function in Drosophila and mice. Curr. Opin. Genet. Dev. *22*, 354–360.
18. Patel, P.H., and Edgar, B.A. (2014). Tissue design: how Drosophila tumors remodel their neighborhood. Semin. Cell Dev. Biol. *28*, 86–95.

19. Cordero, J., Vidal, M., and Sansom, O. (2009). APC as a master regulator of intestinal homeostasis and transformation: from flies to vertebrates. Cell Cycle *8*, 2926–2931.
20. Lee, W.C., Beebe, K., Sudmeier, L., and Micchelli, C.A. (2009). Adenomatous polyposis coli regulates Drosophila intestinal stem cell proliferation. Development *136*, 2255–2264.
21. Patel, P.H., Dutta, D., and Edgar, B.A. (2015). Niche appropriation by Drosophila intestinal stem cell tumours. Nat. Cell Biol. *17*, 1182–1192.
22. Kolahgar, G., Suijkerbuijk, S.J.E., Kucinski, I., Poirier, E.Z., Mansour, S., Simons, B.D., and Piddini, E. (2015). Cell competition modifies adult stem cell and tissue population dynamics in a JAK- STAT-dependent manner. Dev. Cell *34*, 297–309.
23. Vincent, J.-P., Kolahgar, G., Gagliardi, M., and Piddini, E. (2011). Steep differences in wingless signaling trigger Myc-independent competitive cell interactions. Dev. Cell *21*, 366–374.
24. Cordero, J.B., Stefanatos, R.K., Myant, K., Vidal, M., and Sansom, O.J. (2012). Non-autonomous crosstalk between the Jak/Stat and Egfr pathways mediates Apc1-driven intestinal stem cell hyperplasia in the Drosophila adult midgut. Development *139*, 4524–4535.
25. Loberg, R.D., Bradley, D.A., Tomlins, S.A., Chinnaiyan, A.M., and Pienta, K.J. (2007). The lethal phenotype of cancer: the molecular basis of death due to malignancy. CA Cancer J. Clin. *57*, 225–241.
26. Mathur, D., Bost, A., Driver, I., and Ohlstein, B. (2010). A transient niche regulates the specification of Drosophila intestinal stem cells. Science *327*, 210–213.
27. Jiang, H., Patel, P.H., Kohlmaier, A., Grenley, M.O., McEwen, D.G., and Edgar, B.A. (2009). Cytokine/Jak/Stat signaling mediates regeneration and homeostasis in the Drosophila midgut. Cell *137*, 1343–1355.
28. Biteau, B., Hochmuth, C.E., and Jasper, H. (2008). JNK activity in somatic stem cells causes loss of tissue homeostasis in the aging Drosophila gut. Cell Stem Cell *3*, 442–455.
29. Buchon, N., Broderick, N.A., Chakrabarti, S., and Lemaitre, B. (2009). Invasive and indigenous microbiota impact intestinal stem cell activity through multiple pathways in Drosophila. Genes Dev. *23*, 2333–2344.
30. Tamori, Y., and Deng, W.-M. (2011). Cell competition and its implications for development and cancer. J. Genet. Genomics *38*, 483–495.
31. Igaki, T., Pagliarini, R.A., and Xu, T. (2006). Loss of cell polarity drives tumor growth and invasion through JNK activation in Drosophila. Curr. Biol. *16*, 1139–1146.
32. Harvey, K., and Tapon, N. (2007). The Salvador-Warts-Hippo pathway - an emerging tumour-suppressor network. Nat. Rev. Cancer *7*, 182–191.
33. Neto-Silva, R.M., de Beco, S., and Johnston, L.A. (2010). Evidence for a growth-stabilizing regulatory feedback mechanism between Myc and Yorkie, the Drosophila homolog of Yap. Dev. Cell *19*, 507–520.
34. Ziosi, M., Baena-López, L.A., Grifoni, D., Froldi, F., Pession, A., Garoia, F., Trotta, V., Bellosta, P., Cavicchi, S., and Pession, A. (2010). dMyc functions downstream of Yorkie to promote the supercompetitive behavior of hippo pathway mutant cells. PLoS Genet. *6*, e1001140.
35. Chen, C.-L., Schroeder, M.C., Kango-Singh, M., Tao, C., and Halder, G. (2012). Tumor suppression by cell competition through regulation of the Hippo pathway. Proc. Natl. Acad. Sci. USA *109*, 484–489.
36. Azzolin, L., Zanconato, F., Bresolin, S., Forcato, M., Basso, G., Bicciato, S., Cordenonsi, M., and Piccolo, S. (2012). Role of TAZ as mediator of Wnt signaling. Cell *151*, 1443–1456.
37. Konsavage, W.M., Jr., Kyler, S.L., Rennoll, S.A., Jin, G., and Yochum, G.S. (2012). Wnt/β-catenin signaling regulates Yes-associated protein (YAP) gene expression in colorectal carcinoma cells. J. Biol. Chem. *287*, 11730–11739.
38. Holmgren, L., O'Reilly, M.S., and Folkman, J. (1995). Dormancy of micrometastases: balanced proliferation and apoptosis in the presence of angiogenesis suppression. Nat. Med. *1*, 149–153.
39. Grzeschik, N.A., Amin, N., Secombe, J., Brumby, A.M., and Richardson, H.E. (2007). Abnormalities in cell proliferation and apico-basal cell polarity are separable in Drosophila lgl mutant clones in the developing eye. Dev. Biol. *311*, 106–123.
40. Brumby, A.M., and Richardson, H.E. (2003). scribble mutants cooperate with oncogenic Ras or Notch to cause neoplastic overgrowth in Drosophila. EMBO J. *22*, 5769–5779.
41. Martins, V.C., Busch, K., Juraeva, D., Blum, C., Ludwig, C., Rasche, V., Lasitschka, F., Mastitsky, S.E., Brors, B., Hielscher, T., et al. (2014). Cell competition is a tumour suppressor mechanism in the thymus. Nature *509*, 465–470.
42. Snippert, H.J., Schepers, A.G., van Es, J.H., Simons, B.D., and Clevers, H. (2014). Biased competition between Lgr5 intestinal stem cells driven by oncogenic mutation induces clonal expansion. EMBO Rep. *15*, 62–69.
43. Vermeulen, L., Morrissey, E., van der Heijden, M., Nicholson, A.M., Sottoriva, A., Buczacki, S., Kemp, R., Tavaré, S., and Winton, D.J. (2013). Defining stem cell dynamics in models of intestinal tumor initiation. Science *342*, 995–998.
44. Halder, G., Dupont, S., and Piccolo, S. (2012). Transduction of mechanical and cytoskeletal cues by YAP and TAZ. Nat. Rev. Mol. Cell Biol. *13*, 591–600.
45. Huang, J., Wu, S., Barrera, J., Matthews, K., and Pan, D. (2005). The Hippo signaling pathway coordinately regulates cell proliferation and apoptosis by inactivating Yorkie, the Drosophila Homolog of YAP. Cell *122*, 421–434.
46. Varelas, X., Miller, B.W., Sopko, R., Song, S., Gregorieff, A., Fellouse, F.A., Sakuma, R., Pawson, T., Hunziker, W., McNeill, H., et al. (2010). The Hippo pathway regulates Wnt/beta-catenin signaling. Dev. Cell *18*, 579–591.
47. Tanaka, T., Kohno, H., Suzuki, R., Hata, K., Sugie, S., Niho, N., Sakano, K., Takahashi, M., and Wakabayashi, K. (2006). Dextran sodium sulfate strongly promotes colorectal carcinogenesis in Apc(Min/+) mice: inflammatory stimuli by dextran sodium sulfate results in development of multiple colonic neoplasms. Int. J. Cancer *118*, 25–34.
48. Itzkowitz, S.H., and Yio, X. (2004). Inflammation and cancer IV. Colorectal cancer in inflammatory bowel disease: the role of inflammation. Am. J. Physiol. Gastrointest. Liver Physiol. *287*, G7–G17.
49. Chan, A.T., Ogino, S., and Fuchs, C.S. (2009). Aspirin use and survival after diagnosis of colorectal cancer. JAMA *302*, 649–658.
50. Osterwalder, T., Yoon, K.S., White, B.H., and Keshishian, H. (2001). A conditional tissue-specific transgene expression system using inducible GAL4. Proc. Natl. Acad. Sci. USA *98*, 12596–12601.

Current Biology, Volume *26*

Supplemental Information

Cell Competition Drives the Growth of Intestinal Adenomas in *Drosophila*

Saskia J.E. Suijkerbuijk, Golnar Kolahgar, Iwo Kucinski, and Eugenia Piddini

Supplemental Figures and Legends

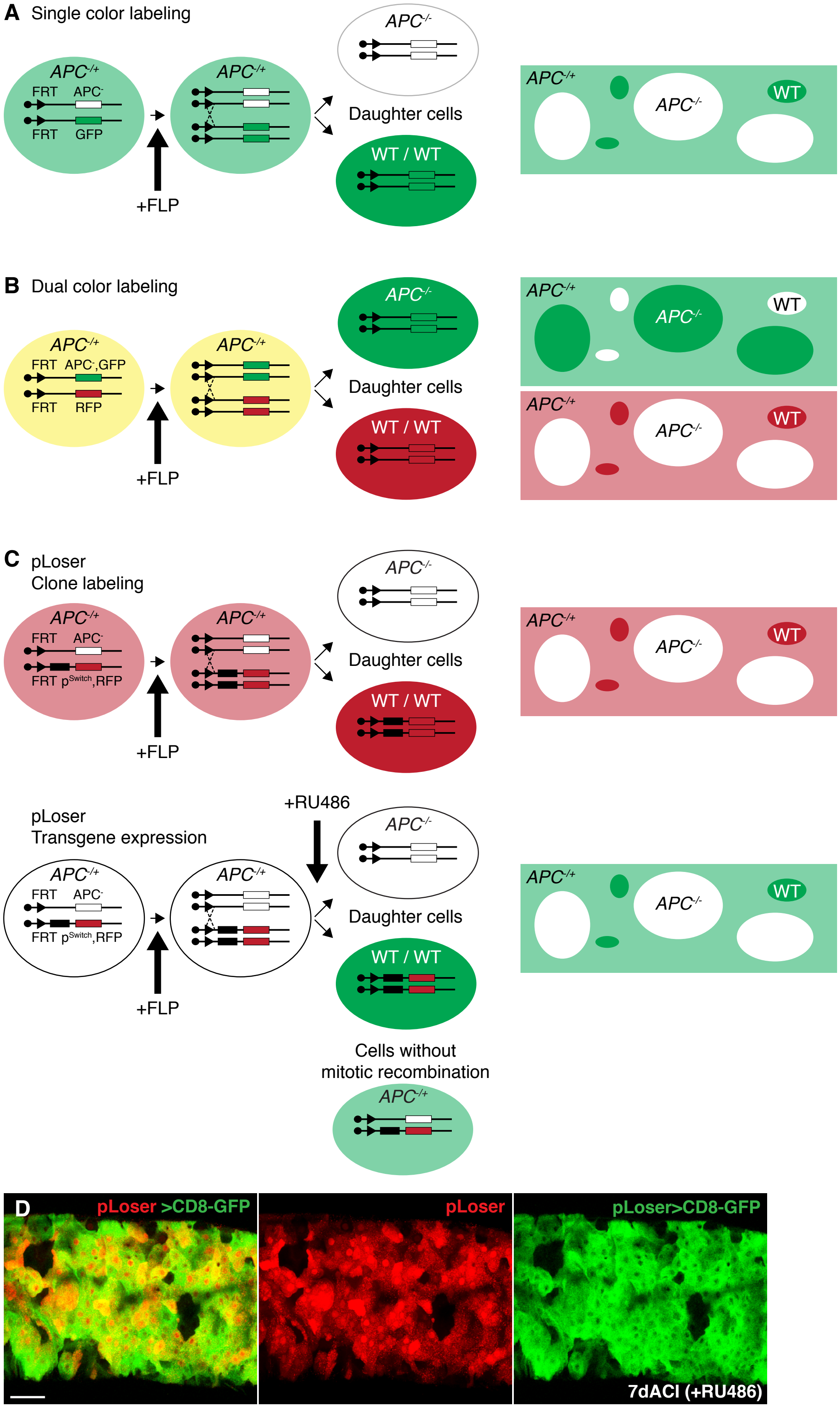

Figure S1, Introduction to single and dual color lineage tracing, Related to Experimental Procedures

A-B) Schematic representation of single (A) and dual (B) color lineage tracing. Heat-shock induced expression of FLP causes mitotic recombination in stem cells that are heterozygous *APC* mutant and express one copy of GFP (A) or one copy of GFP and one copy of RFP (B). This results in the generation of daughter cells that are $APC^{-/-}$ (top) or WT/WT (bottom). Clones of cells that are generated by single color labeling (A) are marked by absence of GFP ($APC^{-/-}$) or two copies of GFP (WT). Clones of cells that are generated by dual color labeling (B) are marked by absence of RFP and two copies of GFP ($APC^{-/-}$) or absence of GFP and two copies of RFP (WT). C) Schematic representation of single color lineage tracing combined with pLoser based transgene expression in host cells. Heat-shock induced expression of FLP causes mitotic recombination in stem cells that are heterozygous *APC* mutant and express one copy of RFP and a RU486 (mifepristone)-inducible GeneSwitch. This results in the generation of daughter cells that are $APC^{-/-}$ (top) or WT/WT (bottom). Clones of cells that are generated by pLoser single color labeling are marked by absence of RFP and lack expression of the GeneSwitch. D) Posterior midguts harboring *hs*-FLP induced WT clones marked by absence of RFP. Expression of CD8-GFP was induced by activation of the pLoser GeneSwitch in cells surrounding clones (200µM RU486). Note that absence of RFP directly correlates to absence of GFP induction.

Genotype:

D) *hs-FLP; UAS-CD8-GFP; FRT82B, pLoser, Ubi-RFP-nls / FRT82B*

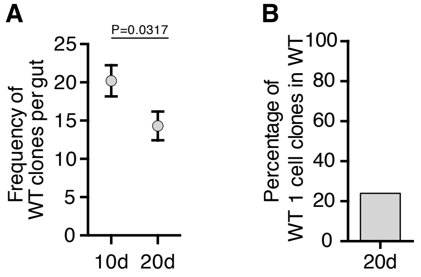

Figure S2, *APC$^{-/-}$*- induced cell competition causes attrition of healthy tissue, Related to Figure 2

A) Frequency of WT clones per midgut from guts containing *APC$^{-/-}$* clones dissected 10d or 20d ACI (left n=231 and right n=187 clones, ±SEM, P-value is displayed above graph, Mann-Whitney test). B) Percentage of 1-cell clones across the whole population of WT clones in control posterior midguts 20d ACI (n=50 clones).

Genotypes:

A) *hs-FLP; pSwitch^{all} /+; FRT82B, APC2^{G10}, APC1^{Q8}, Ubi-GFP/ FRT82B, Ubi-RFP-nls*

B) *hs-FLP;; FRT82B, Ubi-GFP/ FRT82B*

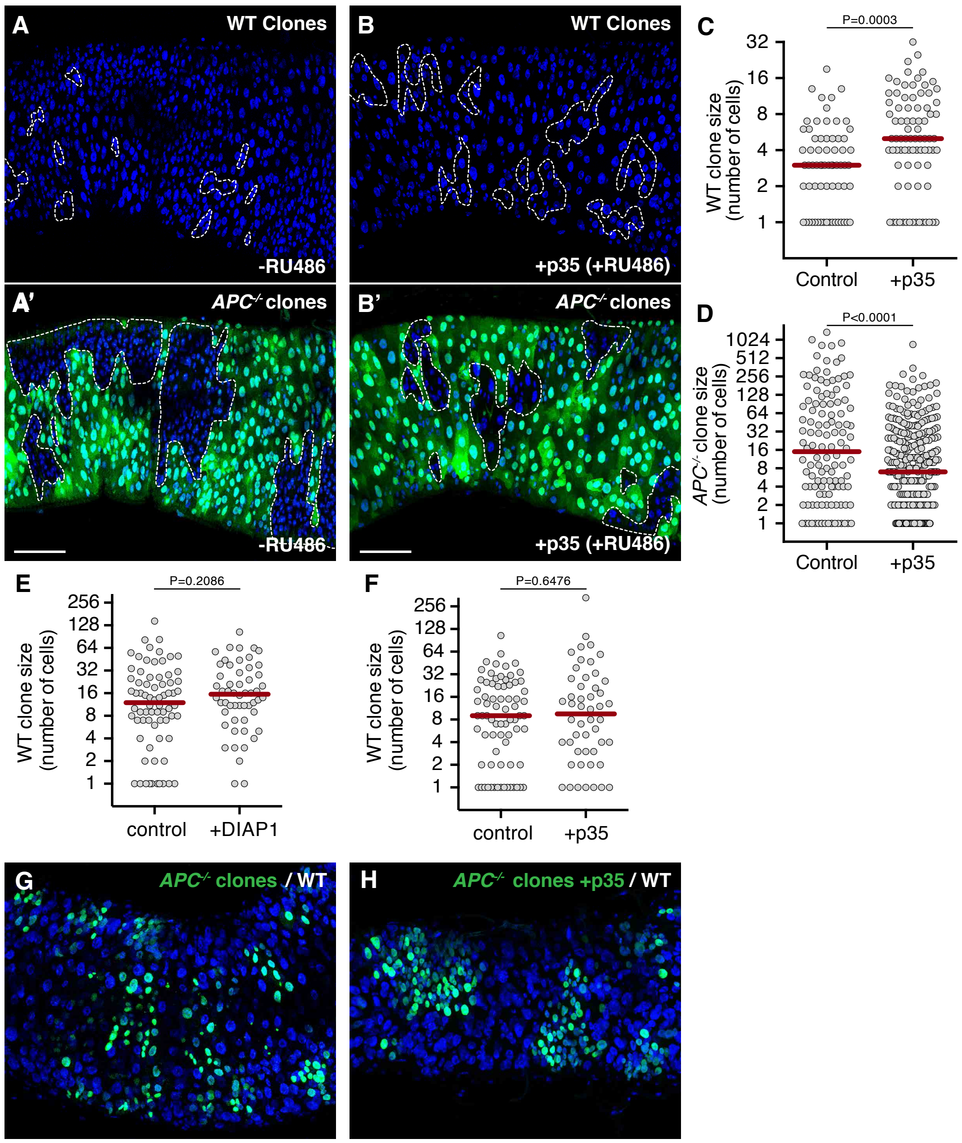

Figure S3, Cell competition fuels tumor growth, Related to Figure 3

A-D) Posterior midguts harboring *hs*-FLP induced WT clones, marked by two copies of GFP (A and B), and *APC*$^{-/-}$ clones, marked by absence of GFP (A' and B') 17dACI. Cell death was blocked by inducible expression of p35 in all ECs and progenitors cells using the GeneSwitch system (+p35, 200μM RU486; B and B'). Control guts (A and A') are of the same genotype as B but were treated with carrier only (-RU486). Graphs in C and D display the distribution of WT (C) or *APC*$^{-/-}$ (D) clone sizes on the y-axis (Log2 scale) from guts with (right) or without (left) p35 expression. Each dot represents one clone and the red bar indicates the median clone size (C left n=67, C right n=82, D left n=122 and D right n=345 clones). E-F) Graphs in E and F display on the y-axis (Log2 scale) the size distribution of *hs*-FLP induced WT clones marked by absence of GFP in WT control posterior midguts (E and F, left graphs) or in WT midguts where cell death was blocked by inducible expression of either DIAP1 (E, right graph) or p35 (F, right graph) in all

ECs and progenitors cells using the GeneSwitch system (40μM RU486). Control guts were treated similarly to experimental guts, but lack the UAS transgene. Each dot represents one clone and the red bar indicates the median clone size (E left n=72, E right n=54, F left n=74 and F right n=50 clones). G-H) Posterior midguts harboring *hs*-FLP induced $APC^{-/-}$ clones marked by MARCM-driven expression of GFP, with (H, p35) or without (G, control) additional expression of the cell death inhibitor p35 exclusively in $APC^{-/-}$ cells at 17dACI. P-values are displayed above graphs (Mann-Whitney test).

Genotypes:

A-D) *hs-FLP; pSwitch^{all}/ UAS-p35; FRT82B, Ubi-GFP/ FRT82B, APC2^{G10}, APC1^{Q8}*

E left) *hs-FLP; pSwitch^{all}/ GlBc; FRT82B, Ubi-GFP/ FRT82B*

E right) *hs-FLP; pSwitch^{all}/ UAS-DIAP1; FRT82B, Ubi-GFP/ FRT82B*

F left) *hs-FLP; pSwitch^{all}/ CyO; FRT82B, Ubi-GFP/ FRT82B*

F right) *hs-FLP; pSwitch^{all}/ UAS-p35; FRT82B, Ubi-GFP/ FRT82B*

G) *hs-FLP,UAS-GFP-nls,Tub-Gal4;; FRT82B, Tub-Gal80/ FRT82B, APC2^{G10}, APC1^{Q8}*

H) *hs-FLP,UAS-GFP-nls,Tub-Gal4; UAS-p35/+; FRT82B,Tub-Gal80/ FRT82B, APC2^{G10}, APC1^{Q8}*

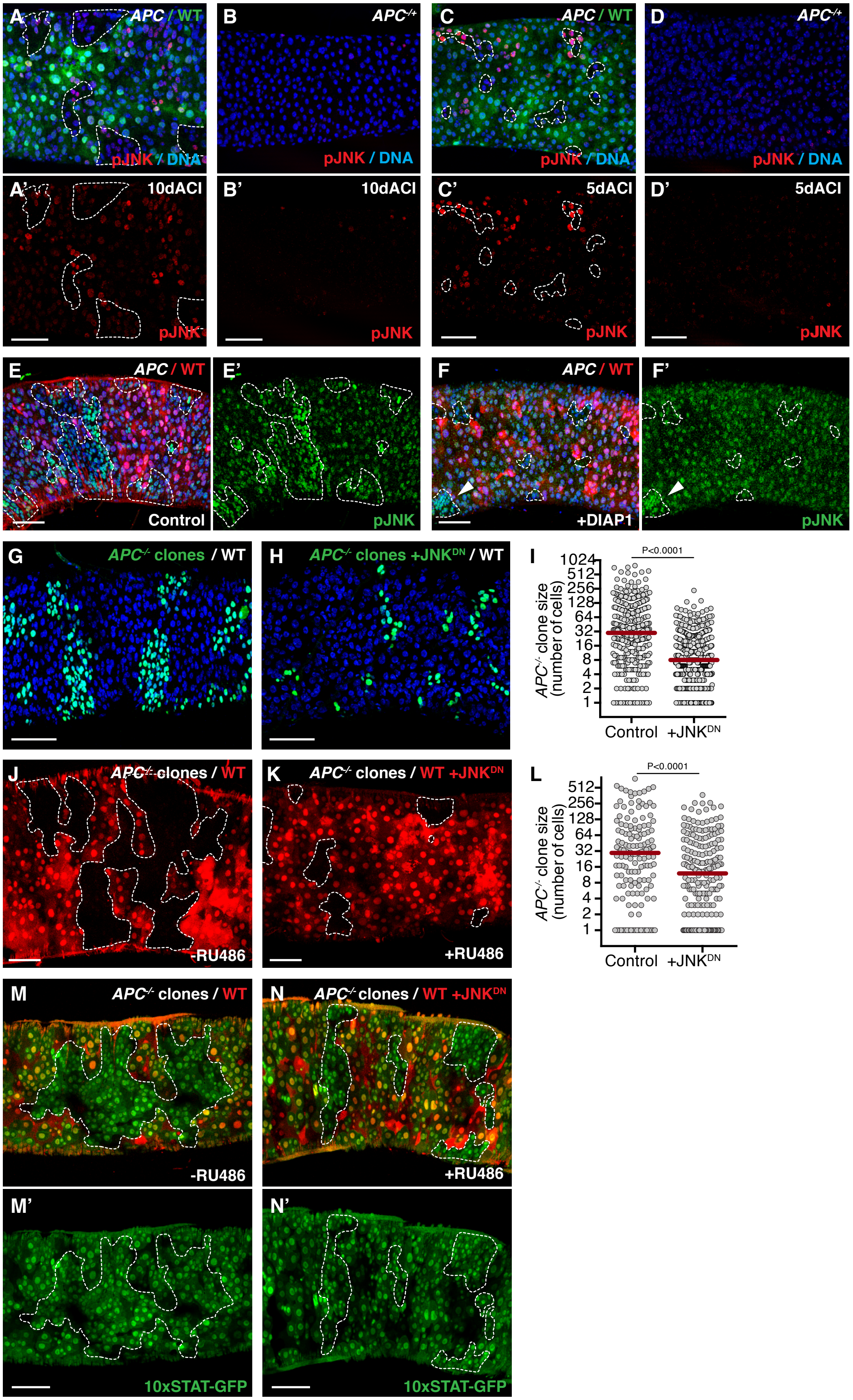
A
APC / WT
pJNK / DNA
B
APC-/+
pJNK / DNA
C
APC / WT
pJNK / DNA
D
APC-/+
pJNK / DNA
A'
10dACI
pJNK
B'
10dACI
pJNK
C'
5dACI
pJNK
D'
5dACI
pJNK
E
APC / WT
Control
E'
pJNK
F
APC / WT
+DIAP1
F'
pJNK
G
APC-/- clones / WT
H
APC-/- clones +JNKDN / WT
I
P<0.0001
APC-/- clone size (number of cells)
1024
512
256
128
64
32
16
8
4
2
1
Control
+JNKDN
J
APC-/- clones / WT
-RU486
K
APC-/- clones / WT +JNKDN
+RU486
L
P<0.0001
APC-/- clone size (number of cells)
512
256
128
64
32
16
8
4
2
1
Control
+JNKDN
M
APC-/- clones / WT
-RU486
N
APC-/- clones / WT +JNKDN
+RU486
M'
10xSTAT-GFP
N'
10xSTAT-GFP

Figure S4, JNK signaling boosts *APC$^{-/-}$* adenoma growth, Related to Figure 4

A-D) Posterior midguts stained with anti-phospho (active) JNK (pJNK, red). B and D display *APC$^{-/+}$* heterozygous guts and A and C guts containing *hs*-FLP induced *APC$^{-/-}$* clones, marked by absence of GFP, dissected 5d (C and D) or 10d (A and B) ACI. E-F) Posterior midguts harboring *hs*-FLP induced *APC$^{-/-}$* clones marked by absence of RFP dissected 17dACI and stained with anti-phospho (active) JNK (pJNK, green). Cell death was blocked by inducible expression of DIAP1 in all ECs and progenitors cells using the GeneSwitch system (F, +DIAP1, 40μM RU486), control guts were treated similarly to experimental guts, but lack the UAS-DIAP1 transgene (E, control). The arrowhead in F points to an *APC$^{-/-}$* clone with higher pJNK staining. G-I) Posterior midguts harboring *hs*-FLP induced *APC$^{-/-}$* clones marked by MARCM-driven expression of GFP, with (H) or without (G) additional expression of JNK^{DN} within the clones at 17dACI. Graph in I displays the distribution of *APC$^{-/-}$* clone sizes for guts of the same genotypes as in G-H (left n=347 and right n=487 clones). J-L) Posterior midguts harboring *hs*-FLP induced *APC$^{-/-}$* clones dissected 17dACI. Clones are marked by absence of RFP. JNK signaling was blocked in K by inducible expression of JNK^{DN} in host cells using the GeneSwitch system (+JNK^{DN}, 40μM RU486). Control guts (J) are of the same genotype as K but were treated with carrier only. Graph in L displays the distribution of *APC$^{-/-}$* clone sizes on the y-axis (Log2 scale) with (right) or without (left) JNK^{DN} expression in host cells. Each dot represents one clone and the red bar indicates the median clone size (left n=148 and right n=214 clones). M-N) Posterior midguts harboring *hs*-FLP induced *APC$^{-/-}$* clones, marked by absence of RFP, dissected 17dACI. JAK/STAT activity was detected by expression of 10xSTAT-GFP (green). JNK signaling was blocked in N by inducible expression of JNK^{DN} in surrounding cells using the GeneSwitch system starting 7d ACI (+JNK^{DN}, 10days 40μM RU486). Control guts (M) are of the same genotype as N but were treated with carrier only. P-values are displayed above graphs (Mann-Whitney test). Genotypes:

A and C)	*hs-FLP;; FRT82B, Ubi-GFP/ FRT82B, APC2^{G10}, APC1^{Q8}*
B and D)	*hs-FLP;; FRT82B, APC2^{G10}, APC1^{Q8}/+*
E)	*hs-FLP ; pSwitch^{all} / CyO ; diap1-LacZ, FRT82B, Ubi-RFP-nls / FRT82B, APC2^{G10}, APC1^{Q8}*
F)	*hs-FLP ; pSwitch^{all} / UAS-DIAP1 ; diap1-LacZ, FRT82B, Ubi-RFP-nls / FRT82B, APC2^{G10}, APC1^{Q8}*
G, I (left)	*hs-FLP, UAS-GFP-nls,Tub-Gal4 ;; FRT82B, Tub-Gal80/ FRT82B, APC2^{G10},APC1^{Q8}*
H, I (right)	*hs-FLP, UAS-GFP-nls, Tub-Gal4 / UAS-Bsk^{DN};; FRT82B, TubGal80/ FRT82B, APC2^{G10}, APC1^{Q8}*
J-L)	*hs-FLP ; UAS-Puc/+ ; FRT82B, pLoser, Ubi-RFP-nls / FRT82B,APC2^{G10},APC1^{Q8}*
M-N)	*hs-FLP / UAS-Bsk^{DN} ; 10xSTAT-GFP/+ ; FRT82B, pLoser, Ubi-RFP-nls / FRT82B, APC2^{G10}, APC1^{Q8}*

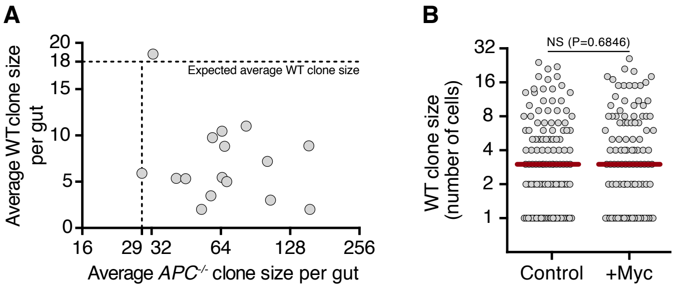

Figure S5, Tumor growth is required for cell competition, Related to Figure 5

A) Graph displaying the average $APC^{-/-}$ clone size per gut (x-axis) plotted against the average WT clone size (y-axis) for clones from guts of the same genotype as in Figure 2B. The dotted horizontal line indicates the expected average WT clone size 17dACI based on the data shown in Figure 2A and 2C, left. The dotted vertical line indicates the extrapolated *APC*$^{-/-}$ clone size that is sufficient to induce competition. B) Graph displaying the size distribution on the y-axis (Log2 scale) of WT clones marked by two copies of RFP from guts containing $APC^{-/-}$ clones with (right) or without (left) additional Myc expression throughout host cells (from guts of the same genotype as in Figures 5K (left) L (right)). Each dot represents one clone and the red bar indicates the median clone size (left n=115 and right n=105 clones). The P-value is displayed above graphs (Mann-Whitney test).

Genotypes:

A) *hs-FLP; pSwitch*^{all} */+; FRT82B, APC2*^{G10}*, APC1*^{Q8}*, Ubi-GFP/ FRT82B, Ubi-RFP-nls*

B) *hs-FLP ; UAS-Myc/+ ; FRT82B, pLoser, Ubi-RFP-nls / FRT82B, APC2*^{G10}*, APC1*^{Q8}

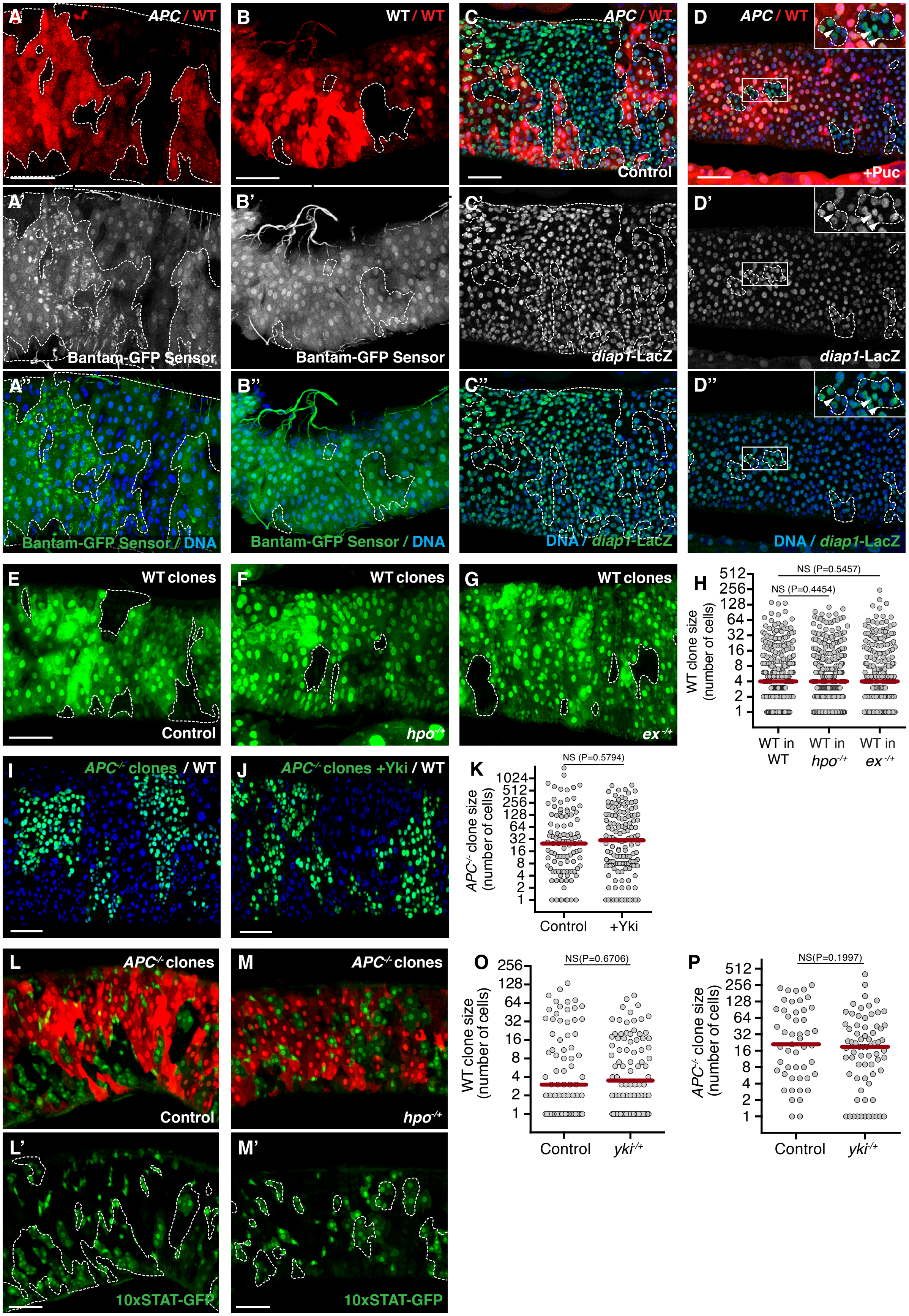

A APC / WT
A' Bantam-GFP Sensor
A'' Bantam-GFP Sensor / DNA
B WT / WT
B' Bantam-GFP Sensor
B'' Bantam-GFP Sensor / DNA
C APC / WT
Control
C' diap1-LacZ
C'' DNA / diap1-LacZ
D APC / WT
+Puc
D' diap1-LacZ
D'' DNA / diap1-LacZ
E WT clones
Control
F WT clones
hpo-/+
G WT clones
ex-/+
H
NS (P=0.5457)
NS (P=0.4454)
WT clone size (number of cells)
512
256
128
64
32
16
8
4
2
1
WT in WT
WT in hpo-/+
WT in ex-/+
I APC-/- clones / WT
J APC-/- clones +Yki / WT
K
NS (P=0.5794)
APC-/- clone size (number of cells)
1024
512
256
128
64
32
16
8
4
2
1
Control
+Yki
L APC-/- clones
Control
L' 10xSTAT-GFP
M APC-/- clones
hpo-/+
M' 10xSTAT-GFP
O
NS(P=0.6706)
WT clone size (number of cells)
256
128
64
32
16
8
4
2
1
Control
yki-/+
P
NS(P=0.1997)
APC-/- clone size (number of cells)
512
256
128
64
32
16
8
4
2
1
Control
yki-/+

Figure S6, Differences in Yki activity determine cell competition potential of *APC*$^{-/-}$ cells, Related to Figure 6

A-B) Posterior midguts with *hs*-FLP induced *APC*$^{-/-}$ (A) or WT (B) clones, marked by absence of RFP, dissected 17d ACI. Yki activity was detected by decreased expression of the Bantam-GFP sensor (A' and B' white and A'' and B'' green). Increased activity was observed in at least one *APC*$^{-/-}$ clone per gut, in about two thirds of *APC*$^{-/-}$ adenoma containing guts (63% n=19). C-D) Posterior midguts harboring *hs*-FLP induced *APC*$^{-/-}$ clones marked by absence of RFP dissected 17dACI. Yki activity was detected by expression of *diap1*-LacZ (C' and D' white and C, D, C'' and D'' green). JNK signaling was blocked by inducible expression of Puckered (D) in all ECs and progenitors cells using the GeneSwitch system (40μM RU486), control guts were treated similarly to experimental guts, but lack UAS transgenes (C, control). Insets in D displays regions containing *APC*$^{-/-}$ clones and arrowheads point at small *APC*$^{-/-}$ mutant cells. E-H) Guts containing WT clones marked by absence of GFP, in control (E), *hpo*$^{-/+}$ (F) or *ex*$^{-/+}$ (G) heterozygous posterior midguts 17dACI. Graph in H displays the distribution of WT clone sizes on the y-axis (Log2 scale) from control (left), *hpo*$^{-/+}$ (middle) or *ex*$^{-/+}$ (right) heterozygous guts (left n=329, middle n=328 and right n=247 clones). I-K) Posterior midguts harboring *hs*-FLP induced *APC*$^{-/-}$ clones marked by MARCM-driven expression of GFP, with (J) or without (I) additional expression of Yki within the clones at 17dACI. Graph in K displays the distribution of *APC*$^{-/-}$ clone sizes for guts of the same genotypes as in I-J (left n=102 and right n=135 clones). L-M) Posterior midguts harboring *hs*-FLP induced *APC*$^{-/-}$ clones, marked by absence of RFP, in a control (L and L') or *hpo*$^{-/+}$ (M and M') heterozygous background, dissected 17dACI. JAK/STAT activity was detected by expression of 10xSTAT-GFP (green). O-P) Graphs display the distribution of simultaneously induced GFP-negative WT (O) or RFP-negative *APC*$^{-/-}$ (P) clone sizes on the y-axis (Log2 scale) from control (left) or *yki*$^{-/+}$ (right) guts. In all graphs each dot represents one clone, the red bars indicate median clone sizes and P-values are displayed above (Mann-Whitney test). Genotypes:

A) *hs-FLP ; Bantam-GFP/ +; FRT82B, Ubi-RFP-nls / FRT82B, APC2^{G10}, APC1^{Q8}*
D) *hs-FLP; Bantam-GFP/+; FRT82B, Ubi-RFP-nls/ FRT82B*
C) hs-FLP ; pSwitch^{all} / CyO ; diap1-LacZ, FRT82B, Ubi-RFP-nls/ FRT82B, APC2^{G10}, APC1^{Q8}
D) hs-FLP; pSwitch^{all}/ UAS-Puc; diap1-LacZ,FRT82B,Ubi-RFP-nls/ FRT82B,APC2^{G10},APC1^{Q8}
E&H) *hs-FLP;; FRT82B, Ubi-RFP-nls/ FRT82B, Ubi-GFP*
F&H) *hs-FLP; hpo$^{42-47}$/+; FRT82B, Ubi-RFP-nls/ FRT82B, Ubi-GFP*
G&H) *hs-FLP; ex^{ex1}/+; FRT82B, Ubi-RFP-nls/ FRT82B, Ubi-GFP*
I& K) *hs-FLP, UAS-GFP-nls,Tub-Gal4; GlBc/+; FRT82B,Tub-Gal80 / FRT82B,APC2^{G10}, APC1^{Q8}*
J& K) *hs-FLP,UAS-GFP-nls,Tub-Gal4;UAS-Yki/+;FRT82B,TubGal80/FRT82B,APC2^{G10},APC1^{Q8}*
L) *hs-FLP ; 10xSTAT-GFP/+ ; FRT82B, Ubi-RFP-nls / FRT82B, APC2^{G10}, APC1^{Q8}*
M) *hs-FLP ; 10xSTAT-GFP/ hpo$^{42-47}$; FRT82B, Ubi-RFP-nls / FRT82B, APC2^{G10}, APC1^{Q8}*
O&P) *hs-FLP; Sp/+; FRT82B, Ubi-RFP-nls / FRT82B, APC2^{G10}, APC1^{Q8}, Ubi-GFP*
O&P *hs-FLP; Yki^{B5}/+; FRT82B, Ubi-RFP-nls / FRT82B, APC2^{G10}, APC1^{Q8}, Ubi-GFP*

Supplemental Experimental Procedures

Experimental Genotypes

Figure 1:

A: *hs-FLP ;; FRT82B, Ubi-GFP / FRT82B*
B: *hs-FLP ;; FRT82B, Ubi-GFP / FRT82B, APC2*^{G10}*, APC1*^{Q8}
D, C (left): *hs-FLP ; pSwitch*^{all} */+; FRT82B, Ubi-GFP / FRT82B, Ubi-RFP-nls*
E, C (right): *hs-FLP; pSwitch*^{all} */+; FRT82B, APC2*^{G10}*, APC1*^{Q8}*, Ubi-GFP / FRT82B, Ubi-RFP-nls*
F, H (left): *hs-FLP ;; FRT82B, Act-Gal4, UAS-CD8-hPARP-Venus / FRT82B*
G,H (right),I: *hs-FLP;; FRT82B, Act-Gal4, UAS-CD8-hPARP-Venus / FRT82B, APC2*^{G10}*, APC1*^{Q8}

Figure 2:

A, C (left): *hs-FLP ; pSwitch*^{all} */+; FRT82B, Ubi-GFP / FRT82B, Ubi-RFP-nls*
B, C(right),D: *hs-FLP ; pSwitch*^{all} */+; FRT82B, APC2*^{G10}*,APC1*^{Q8}*, Ubi-GFP / FRT82B, Ubi-RFP-nls*

Figure 3:

A-D: *hs-FLP ; pSwitch*^{all} */ UAS-DIAP1 ; FRT82B, Ubi-GFP / FRT82B, APC2*^{G10}*, APC1*^{Q8}
E, G (left): *hs-FLP, UAS-GFP-nls, Tub-Gal4 ; GlBc / + ; FRT82B, Tub-Gal80 / FRT82B, APC2*^{G10}*, APC1*^{Q8}
F, G (right): *hs-FLP,UAS-GFP-nls,Tub-Gal4 ; UAS-DIAP1/+ ; FRT82B,Tub-Gal80 / FRT82B,APC2*^{G10}*,APC1*^{Q8}
H-J: *hs-FLP ; UAS-DIAP1/+ ; FRT82B, pLoser, Ubi-RFP-nls / FRT82B,APC2*^{G10}*,APC1*^{Q8}

Figure 4:

A: *hs-FLP ;; FRT82B, Ubi-GFP / FRT82B*
B: *hs-FLP ;; FRT82B, Ubi-GFP / FRT82B, APC2*^{G10}*, APC1*^{Q8}
C-F: *hs-FLP ; pSwitch*^{all} */ UAS-Puc ; FRT82B, Ubi-GFP / FRT82B, APC2*^{G10}*, APC1*^{Q8}
G, I& J (left): *hs-FLP, UAS-GFP-nls,Tub-Gal4;; FRT82B, Tub-Gal80 / FRT82B, APC2*^{G10}*, APC1*^{Q8}*, Ubi-GFP*
H, I& J(right): *hs-FLP, UAS-GFP-nls, Tub-Gal4 ; UAS-Puc/ +; FRT82B, TubGal80 / FRT82B, APC2*^{G10}*, APC1*^{Q8}*, Ubi-GFP*
K-M: *hs-FLP ; UAS-Puc/+ ; FRT82B, pLoser, Ubi-RFP-nls / FRT82B,APC2*^{G10}*,APC1*^{Q8}

Figure 5:

A-D: *hs-FLP; pSwitch*^{all} */+; FRT82B, APC2*^{G10}*, APC1*^{Q8}*, Ubi-GFP / FRT82B, Ubi-RFP-nls*
E, G (left): *hs-FLP, UAS-GFP-nls,Tub-Gal4 ;; FRT82B, Tub-Gal80 / FRT82B, APC2*^{G10}*, APC1*^{Q8}*, Ubi-GFP*
F, G (right): *hs-FLP,UAS-GFP-nls,Tub-Gal4; UAS-Puc/+; FRT82B,TubGal80 / FRT82B, APC2*^{G10}*, APC1*^{Q8}*,Ubi-GFP*
H, J (left): *hs-FLP,UAS-GFP-nls,Tub-Gal4 ; GlBc / +; FRT82B,Tub-Gal80 / FRT82B,APC2*^{G10}*,APC1*^{Q8}*,Ubi-GFP*
I, J (right): *hs-FLP, UAS-GFP-nls, Tub-Gal4; UASMyc*^{RNAi} */ +; FRT82B,TubGal80 / FRT82B,APC2*^{G10}*,APC1*^{Q8}*,Ubi-GFP*
K-M: *hs-FLP ; UAS-Myc/+ ; FRT82B, pLoser, Ubi-RFP-nls / FRT82B, APC2*^{G10}*, APC1*^{Q8}

Figure 6:

A: *hs-FLP;; diap1-LacZ, FRT82B, Ubi-RFP-nls / FRT82B, APC2*^{G10}*, APC1*^{Q8}*, Ubi-GFP*
B: *hs-FLP ; pSwitch*^{all} */ UAS-DIAP1 ; diap1-LacZ, FRT82B, Ubi-RFP-nls / FRT82B, APC2*^{G10}*, APC1*^{Q8}
C, F&G(left): *hs-FLP ;; FRT82B, Ubi-RFP-nls / FRT82B, APC2*^{G10}*, APC1*^{Q8}*, Ubi-GFP*
D, F&G(mid): *hs-FLP ; hpo*$^{42-47}$*/ + ; FRT82B, Ubi-RFP-nls / FRT82B, APC2*^{G10}*,APC1*^{Q8}*,Ubi-GFP*
E, F&G(right): *hs-FLP ; ex*^{ex1}*/ + ; FRT82B, Ubi-RFP-nls / FRT82B, APC2*^{G10}*, APC1*^{Q8}*, Ubi-GFP*

Drosophila Stocks

The following fly stocks were used: *hs-FLP;; FRT82B, Ubi-GFP/TM6B, hs-FLP; Sp/CyO; FRT82B, Ubi-RFP-nls/ TM6B* (Daniel StJohnston), *hs-FLP; Sp/CyO; FRT82B, Ubi-GFP/ TM6B, FRT82B (Bloomington), FRT82B, APC2*^{G10}*, APC1*^{Q8}*/TM6B* (M.Peifer), *FRT82B, APC2*^{G10}*, APC1*^{Q8} *, Ubi-GFP/ TM6B* (recombinant generated for this study), *FRT82B, Act-Gal4, UAS-CD8-hPARP-Venus* (recombinant generated for this study from *Act-Gal4, UAS-mCD8-hPARP-Venus* [S1, S2] and *FRT82B*), *pSwitch*^{all} (Recominant generated from *pSwitch*^{AMP} and *pSwitch*^{PC} [S3], previously described [S1], *hs-FLP, UAS-GFP-nls, Tub-Gal4;; FRT82B, Tub-Gal80 / TM6B, w; UAS-DIAP1/Cyo KrGal4, UAS-GFP; TM2/ TM6, Df YFP* ([S4] P. Meier), *UAS-Puc*^{14C} (E. Martin-Blanco [S5]), *UAS-Bsk*^{DN} (E. Martin-Blanco [S6]), *Bantam-GFP / CyO* (S. Cohen [S7]), *diap1-LacZ, FRT82B, Ubi-RFP-nls* (Recombinant generated for this study from *LacZ-diap1*^{j5C8} (Bloomington [S8, S9]) and *FRT82B, Ubi-RFP-nls*), *UAS-p35 ; TM2 / TM6B* [S10], *FRT42D, hpo*$^{42\text{-}47}$*/ CyO* (I. Palacios [S9]), *FRT42D, yki*^{B5}*/ CyO* (I. Palacios [S8]), *w; UAS-yki.GFP*$^{4\text{-}12\text{-}1}$ (Bloomington), *w; FRT40A, ex*^{ex1} */ CyO-GFP; hs-FLP, MKRS / TM6B* (J.P. Vincent [S11]), *UAS-myc* (L.A. Baena-Lopez), *UAS-myc*^{RNAi} (VDRC), 10xSTAT-GFP (E. Bach [S12]). The pLoser line (Figure S1C) was generated by recombination of P{Switch2}GSG2326 (Bloomington) and *FRT82B, Ubi-RFP-nls.* The integration site of P{Switch2}GSG2326 was mapped to chromosome 3R by inverse PCR (www.fruitfly.org/about/methods/inverse.pcr) and induction of expression in the midgut epithelium upon RU486 (mifepristone) feeding was validated with UAS-CD8-mGFP (Figure S1D).

Antibodies:

Mouse anti-Delta (DSHB, C594.9B) 1/1000, mouse anti-Prospero (DSHB, MR1A) 1/50, chicken anti-GFP (Abcam, ab13970) 1/500, rabbit or mouse anti-Cleaved human PARP (Abcam ab2317, 1/100 and Abcam ab110315, 1/500 respectively), rabbit anti-pJNK pTPpY (Promega V793B) 1/500, chicken anti-β-galactosidase (Abcam ab9361) 1/500 and rabbit anti-phospho Histone H3 Ser10 (Cell Signaling 9701) 1/1000. Secondary antibodies used were coupled to Alexa488, Alexa555 or Alexa633 or Cy5 (Molecular Probes). Nuclei were counterstained with DAPI or Hoechst 33342.

Generation of mitotic clones

For clone generation, single stem cell-derived clones were generated by mitotic recombination, using the FLP/FRT system [S13]. One to two days after eclosion, fertilized female flies were heat-shocked in a water bath at 37°C for 10 minutes and then reared at 25°C. Clones were induced

sparsely to minimize clone fusion, except for Figure 6A, where flies were heat-shocked in a water bath at 37°C for 30 minutes, with the intention of generating large clones. Flies were aged up to a maximum of 20 days ACI, to avoid ageing effects, which disrupt tissue homeostasis.

Confocal Acquisition and image analysis

Samples were imaged with Leica SP5 inverted or Leica SP8 upright confocal microscopes, using a 40x 1.3 NA PL Apo or 40x/1.3 HC PL Apo CS2 Oil objective respectively. All images were taken as z-stacks of 1μm sections in the posterior midgut region immediately anterior to the hindgut (these corresponds to the regions P4 in [S14] or region R5 in [S15]. Image processing, analysis and 3D reconstruction were done with Volocity (Perkin Elmer, version 6.3) and Photoshop (Adobe version CS6).

Cell counting

All quantifications were done throughout the volume of 3D reconstructions of z-stacks using Volocity (Perkin Elmer, version 6.3). Quantifications of cell numbers were done manually. Clone sizes were calculated as the number of DAPI positive cells per clone in the 3D volume. To count cells around clones ("near" in Figure 1H) we counted all cells surrounding a clone within two cell diameters in the 3D volume. To characterize cells not adjacent to clones ("far" in Figure 1FH, we counted cells that were not in within two cell diameters of a clone or at the edge of the image. For quantification of WT clones in the absence of tumors (Figure 5F and 5G, right graph) we only included samples in which inhibition of JNK signaling had efficiently reduced tumor growth.

Statistical tests

Statistical analyses were done using Prism (GraphPad, version 6.0 for Mac OS X). P-values were determined using the non-parametric Mann-Whitney test throughout, except for Figures 2D, 4I-J where a Fisher's exact contingency test or t test was used respectively.

Supplemental References

S1. Kolahgar, G., Suijkerbuijk, S. J. E., Kucinski, I., Poirier, E. Z., Mansour, S., Simons, B. D., and Piddini, E. (2015). Cell competition modifies adult stem cell and tissue population dynamics in a JAK- STAT dependent manner. Developmental Cell, 1–14.

S2. Williams, D. W., Kondo, S., Krzyzanowska, A., Hiromi, Y., and Truman, J. W. (2006). Local caspase activity directs engulfment of dendrites during pruning. Nat Neurosci *9*, 1234–1236.

S3. Mathur, D., Bost, A., Driver, I., and Ohlstein, B. (2010). A transient niche regulates the specification of Drosophila intestinal stem cells. Science *327*, 210–213.

S4. Hay, B. A., Wassarman, D. A., and Rubin, G. M. (1995). Drosophila homologs of baculovirus inhibitor of apoptosis proteins function to block cell death. Cell *83*, 1253–1262.

S5. Martín-Blanco, E., Gampel, A., Ring, J., Virdee, K., Kirov, N., Tolkovsky, A. M., and Martinez-Arias, A. (1998). puckered encodes a phosphatase that mediates a feedback loop regulating JNK activity during dorsal closure in Drosophila. Genes Dev. *12*, 557–570.

S6. Adachi-Yamada, T., Nakamura, M., Irie, K., Tomoyasu, Y., Sano, Y., Mori, E., Goto, S., Ueno, N., Nishida, Y., and Matsumoto, K. (1999). p38 mitogen-activated protein kinase can be involved in transforming growth factor beta superfamily signal transduction in Drosophila wing morphogenesis. Molecular and Cellular Biology *19*, 2322–2329.

S7. Brennecke, J., Hipfner, D. R., Stark, A., Russell, R. B., and Cohen, S. M. (2003). bantam encodes a developmentally regulated microRNA that controls cell proliferation and regulates the proapoptotic gene hid in Drosophila. Cell *113*, 25–36.

S8. Huang, J., Wu, S., Barrera, J., Matthews, K., and Pan, D. (2005). The Hippo signaling pathway coordinately regulates cell proliferation and apoptosis by inactivating Yorkie, the Drosophila Homolog of YAP. Cell *122*, 421–434.

S9. Wu, S., Huang, J., Dong, J., and Pan, D. (2003). hippo encodes a Ste-20 family protein kinase that restricts cell proliferation and promotes apoptosis in conjunction with salvador and warts. Cell *114*, 445–456.

S10. Hay, B. A., Wolff, T., and Rubin, G. M. (1994). Expression of baculovirus P35 prevents cell death in Drosophila. Development *120*, 2121–2129.

S11. Hamaratoglu, F., Willecke, M., Kango-Singh, M., Nolo, R., Hyun, E., Tao, C., Jafar-Nejad, H., and Halder, G. (2006). The tumour-suppressor genes NF2/Merlin and Expanded act through Hippo signalling to regulate cell proliferation and apoptosis. Nature Cell Biology *8*, 27–36.

S12. Bach, E. A., Ekas, L. A., Ayala-Camargo, A., Flaherty, M. S., Lee, H., Perrimon, N., and Baeg, G.-H. (2007). GFP reporters detect the activation of the Drosophila JAK/STAT pathway in vivo. Gene Expr. Patterns *7*, 323–331.

S13. Xu, T., and Rubin, G. M. (1993). Analysis of genetic mosaics in developing and adult Drosophila tissues. Development *117*, 1223–1237.

S14. Marianes, A., and Spradling, A. C. (2013). Physiological and stem cell compartmentalization within the Drosophila midgut. Elife *2*, e00886.

S15. Buchon, N., Osman, D., David, F. P. A., Fang, H. Y., Boquete, J.-P., Deplancke, B., and Lemaitre, B. (2013). Morphological and Molecular Characterizationof Adult Midgut Compartmentalization in Drosophila. Cell Rep *3*, 1725–1738.
